# Genomic diversity and population structure of the indigenous Greek and Cypriot cattle populations

**DOI:** 10.1186/s12711-020-00560-8

**Published:** 2020-07-29

**Authors:** Dimitris Papachristou, Panagiota Koutsouli, George P. Laliotis, Elisabeth Kunz, Maulik Upadhyay, Doris Seichter, Ingolf Russ, Bunevski Gjoko, Nikolaos Kostaras, Iosif Bizelis, Ivica Medugorac

**Affiliations:** 1 Laboratory of Animal Husbandry, Faculty of Animal Sciences, Department of Animal Production, Agricultural University of Athens, 75 Iera Odos, 11855 Athens, Greece; 2 Population Genomics Group, Department of Veterinary Sciences, LMU Munich, Lena-Christ-Str. 48, 82152 Martinsried, Germany; 3 Tierzuchtforschung e.V. München, Senator-Gerauer-Str. 23, 85586 Poing, Germany; 4 Livestock Department, Faculty of Agricultural Sciences and Food Institute of Animal Biotechnology, University Ss. Cyril and Methodius, 16-ta Makedonska Brigada 3, 1000 Skopje, North Macedonia; 5 AMALTHIA, Network for the Protection of Greek Indigenous Farm Animals, 51 Argyrokastrou, 15669 Athens, Greece

## Abstract

**Background:**

The indigenous cattle populations from Greece and Cyprus have decreased to small numbers and are currently at risk of extinction due to socio-economic reasons, geographic isolation and crossbreeding with commercial breeds. This study represents the first comprehensive genome-wide analysis of 10 indigenous cattle populations from continental Greece and the Greek islands, and one from Cyprus, and compares them with 104 international breeds using more than 46,000 single nucleotide polymorphisms (SNPs).

**Results:**

We estimated several parameters of genetic diversity (e.g. heterozygosity and allelic diversity) that indicated a severe loss of genetic diversity for the island populations compared to the mainland populations, which is mainly due to the declining size of their population in recent years and subsequent inbreeding. This high inbreeding status also resulted in higher genetic differentiation within the Greek and Cyprus cattle group compared to the remaining geographical breed groups. Supervised and unsupervised cluster analyses revealed that the phylogenetic patterns in the indigenous Greek breeds were consistent with their geographical origin and historical information regarding crosses with breeds of Anatolian or Balkan origin. Cyprus cattle showed a relatively high indicine ancestry. Greek island populations are placed close to the root of the tree as defined by Gir and the outgroup Yak, whereas the mainland breeds share a common historical origin with Buša. Unsupervised clustering and D-statistics analyses provided strong support for *Bos indicus* introgression in almost all the investigated local cattle breeds along the route from Anatolia up to the southern foothills of the Alps, as well as in most cattle breeds along the Apennine peninsula to the southern foothills of the Alps.

**Conclusions:**

All investigated Cyprus and Greek breeds present complex mosaic genomes as a result of historical and recent admixture events between neighbor and well-separated breeds. While the contribution of some mainland breeds to the genetic diversity pool seems important, some island and fragmented mainland breeds suffer from a severe decline of population size and loss of alleles due to genetic drift. Conservation programs that are a compromise between what is feasible and what is desirable should focus not only on the still highly diverse mainland breeds but also promote and explore the conservation possibilities for island breeds.

## Background

Cattle is a species that is present worldwide and well adapted to diverse environments, and has evolved into the most important species for production purposes since its domestication. The presence of cattle was invaluable in the evolution of human societies with a great impact on agricultural development, diet alterations, cultural heritage and social structure [1, 2]. Molecular evidence [3] has indicated two domestication events in cattle from which the taurine (*Bos taurus*) and indicine (*Bos indicus*) species arose, sharing the aurochs (*Bos primigenius*) as common ancestor ~ 250,000 years ago [4]. During the introduction of domesticated cattle into Europe, the Mediterranean coast played a crucial role [2, 5]. Therefore, Greece and Cyprus, which are closely located to the core region of the domestication of cattle, i.e. in the near East, represented an important crossroad for the dispersion of human groups and their herds from Anatolia towards Europe [6–8].

Historically, the Southern Balkan peninsula has been characterized by the free movement of humans and animals, especially in the areas near the current borders, from almost the Neolithic throughout Antiquity, the Roman, Byzantine and Ottoman empire times to nearly 40 years before the present [6, 9]. Pastoralism, the seasonal movement of herds and people to exploit the grazing lands across the Balkan region, is a common practice since centuries [10, 11]. Migration events that enhanced the gene flow among the domesticated cattle populations, genetic drift, physical isolation due to geographic barriers [12] during the above historic periods, and human weak selection pressures led to the formation of well-adapted local cattle breeds in rather marginal and harsh environments [6, 13].

The farming and breeding practices associated with these local breeds differ substantially from those in high-performing breeds [14]: (i) they are fairly undifferentiated and unselected; (ii) pedigree records are incomplete or do not exist; (iii) breeding associations either do not exist or are recently established for conservation purposes only; (iv) accordingly, there are no classical breed standards but a breed is defined by common origin; (v) systematic and standardized records of traits do not exist; and (vi) the infrastructure necessary for such recording is rudimentary. Therefore, these local indigenous populations of South East European cattle do not meet all the conditions to be referred as breeds. However, to avoid confusion between the terms breed, strain and population, we will use the term ‘breed’ only with the adjectives ‘indigenous’, ‘local’ or ‘rare’ or without an adjective. These local breeds have been considered to be under constant risk mainly since the 1970s and onwards. In fact, in some extreme cases, the current population size of some local breeds consists of only a few animals. This global trend [15–17] reflects also, in generic terms, the situation of Greek local breeds.

In Greece and Cyprus, economic and social conditions as well as geomorphological and climatic reasons have not allowed the development of high-yield local cattle populations since 1950. After the 1960s, with the implementation of artificial insemination, many indigenous populations were crossbred with highly selected commercial breeds [18, 19]. For example, Brachyceros, which is a Greek autochthonous breed with short horns resembling the Albanian and Buša cattle, was crossed with Swiss Brown in some regions [20]. In the middle of the 20th century, eight indigenous cattle breeds were reported in Greece. Nowadays, four of these are considered as officially extinct (Tinos, Andros, Chios, Kerkyra), three as threatened (Brachyceros, Katerini and Sykia) and one (Kea) as a rare breed [21, 22]. The aforementioned breeds were mainly bred in mountain regions and/or on islands with poor infrastructure. The latter combined with geographical and natural barriers as well as the absence of artificial insemination led to reproductive isolation, fragmentation, and gradual depletion of the genetic diversity in these breeds [23].

Genetic diversity studies during the past years have been conducted in a large number of domestic cattle breeds [13, 24–30]. Improving our knowledge on the genetic diversity within and among local breeds is considered an issue of crucial importance for enhancing their efficient use with regard to sustainable animal farming in a harsh and less intensified environment, and for implementing further conservation programs [31]. Since the indigenous breeds exhibit adaptive ability to their local environment and remarkable longevity, the genetic pool of unselected local breeds can represent a valuable source of genes [32]. However, with a few exceptions [33, 34], little research has been carried out on the genetic diversity and genetic relationships of indigenous cattle from South East Europe with regard to Greece and Cyprus.

Thus, using the single nucleotide polymorphism (SNP) array technology, our objectives were: (i) to obtain unbiased estimations of the neutral genetic diversity of the Greek cattle populations, which represents the first comprehensive genome-wide analysis for these populations; (ii) to evaluate within-breeds’/populations’ different sources of genetic variance as well as their distinctiveness level; (iii) to predict recent admixture patterns of the highly selected and competitive breeds with the non-selected and heterogeneous indigenous breeds from Greece and Cyprus; (iv) to predict historical admixture patterns in cattle breeds in Greece and their expansion route towards the southern foothills of the Alps; and (v) to build an objective basis for the implementation of conservation programs for breeder’s associations, and national and international bodies, as a solution towards the uncontrolled mating and outcrossing of certain rare breeds under high risk of extinction.

## Methods

### Sampling

**Fig. 1:**
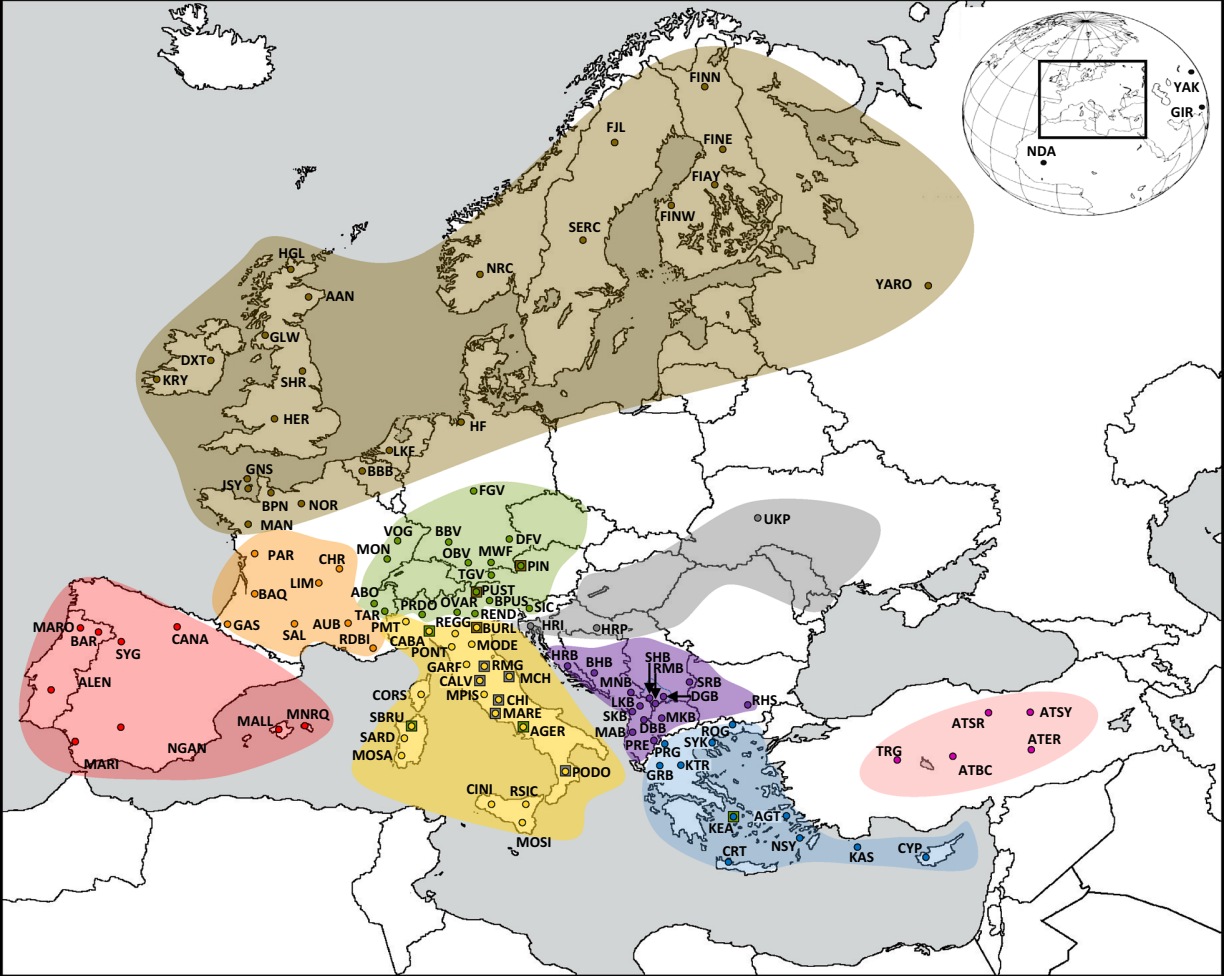
Origin of the breeds used in the analyzed dataset. Special square marks represent the influence of East-Podolian (grey), Alpine (green) and North-West (olive green) groups

Hair roots or blood were sampled from 285 individuals originating from different indigenous cattle populations. Sampling areas are in Fig. 1, which as all subsequent figures, uses the same color code specified for each geographical group in Additional file 1 Table S1. All the samples were collected by trained personnel according to the best veterinary practices. An ethical permission was given by the ethics committee of the Agricultural University of Athens. Briefly, the following local breeds from Greece and Cyprus were sampled in our analysis: (i) from mainland Greece: Greek Brachyceros breed (GRB; n = 97), Katerini breed (KTR; n = 20), Prespa cattle (PRG; n = 10), Rodope cattle (ROG; n = 12), Sykia breed (SYK; n = 16), (ii) from the islands: Kea breed (KEA; n = 97), Agathonisi cattle (AGT; n = 6), Crete cattle (CRT; n = 11), Kastelorizo cattle (KAS; n = 4), Nisyros cattle (NSY; n = 7) and Cyprus cattle (CYP; n = 5). A detailed description of the local cattle breeds studied here is provided in Additional file 2 and see Additional file 1: Table S2. The samples collected in this study were complemented by whole-genome genotypes for GRB (n = 19) and CYP (n = 9) reported by Flori et al. [34] and SYK cattle (n = 5) reported by Verdugo et al. [35] (see Additional file 1: Table S1). In addition, for comparison purposes, we included genetic information of 104 international breeds, based on genetic, historical and geographical criteria. More precisely the large dataset of the above-selected breeds fell into eight main geographic groups (Minor Asia, South East Europe, East Podolian, Tyrrhenian (Apennin-Sicily-Sardinia-Corse), Alpine, France, Iberian and North West European breeds) plus one outgroup that included Gir (GIR), Yak (YAK), and N’Dama (NDA). We included all the above breeds in our study because the geographical origin of some of them have an obvious proximity to Greece and Cyprus or because they have more or less affected the genetic pool of local breeds from Greece and Cyprus through long-term crossbreeding events during the past [36] and possibly till present. For example, the long-term crossing of the indigenous short horned (GRB) cattle and some breeds from the Alpine group (e.g. OBV, BBV and TGV) led to the formation of the KEA breed. However, the GRB and the short horned Buša cattle sampled in the neighboring Balkan countries (South East European group) probably share the same origin, which is why we sampled 18 additional Buša cattle individuals from North Macedonia (see Additional file 1 Table S1). The KTR and SYK breeds are hypothesized to share ancestry with the East Podolian steppe geographic cattle group. The AGT and NSY breeds are hypothesized to share ancestry with some strains of Podolian steppe and Anatolian origin, whereas CYP and KAS might share Anatolian and zebu origins. In addition to the *Bos taurus* breeds that come from Europe and Minor Asia, in our phylogenetic analyses, we used as outgroups YAK from Mongolia, NDA from West Africa representing African *Bos taurus* and GIR cattle originating from India but bred in Brazil, representing *Bos indicus* cattle. These 115 breeds and 10 geographical breed groups used in our analyses are described in Additional file 1: Table S1. We believe that the best way to display the grouping of breeds is by geographical origin (color code) without taking previous genetic knowledge into account, and to improve visualization, we added a symbol for some breeds to indicate possible or known genetic similarities with other breeds or groups. More specifically, these symbols combine the color of the group from which they originate geographically with the color of the group with which they have some genetic similarity. Thus, the breeds BURL (from the Tyrrhenian group), and PUST and PIN (from the Alpine group) display an additional symbol with a color that indicates a known influence from the North West group, the breeds KEA (from Greece), and CABA, AGER, and SBRU (from the Tyrrhenian group) display an additional color that indicates influence from the Alpine group, and some local breeds from Italy (RMG, MCH, CALV, CHI, MARE, and PODO) display an additional color that indicates possible podolian influence [37].

### DNA isolation and SNPs

DNA isolation was performed using a commercial kit (QIAamp DNA MiniKit, QIAGEN) according to the manufacturer’s instructions. For SNP genotyping, the Illumina BovineSNP50 BeadChip array was used following standard procedures (http://www.illumina.com). Furthermore, quality controls were applied to obtain a high-quality dataset: (i) only the animals with call rate higher than 0.95 were included; (ii) SNPs that mapped to unknown or sex chromosomes were removed; (iii) SNPs that were genotyped for less than 90% of the samples were removed; (iv) SNPs with a MAF lower than 0.025 and showing departures from Hardy–Weinberg equilibrium within breed (*P *≤0.01) were removed. The dataset, at this stage, consisted of 46,678 autosomal SNPs genotyped in 3457 individuals.

### Haplotyping and unified additive relationships (UAR)

The Beagle software package (v 5.0) was used for imputation of missing genotypes [38] and haplotype phasing [39]. To improve the efficiency of phasing and imputation, we considered genotyping data of all available bovine animals in the in-house database, which includes a large number of pairs and trios from other projects. Genome-wide relationships between individuals were estimated using a unified additive relationship (UAR) matrix [40] implemented in the R package snpReady [41] and applied to 46,678 SNP genotypes of 3457 animals.

Diversity, phylogeny and population structure analyses require samples of representative and least related animals in each breed. To retain the most representative animals, we excluded outlier individuals (i.e. erroneously sampled and/or admixed animals) by multivariate outlier analysis [42], and then decreased the level of relationship by successive exclusion of highly related animals. Both the multivariate outlier analysis and the reduction of familial structures within breeds rely on the genome-wide additive genetic relationships stored in the UAR matrix. Finally, the dataset used in subsequent diversity and phylogenetic analyses included the 2858 most representative and unrelated animals. The starting and optimized sample sizes for each breed are listed in columns *N* and *Nd* in Additional file 1: Table S1.

### Haplotype diversity

To design the Illumina Bovine SNP50 BeadChip (Illumina), only five taurine breeds and one indicine breed were considered [43]. Therefore, since this array could contain biased sets of pre-ascertained SNPs, we adopted a 4-SNP-block approach as described previously to reduce this ascertainment bias [13, 14]. Specifically, 4-SNP blocks (haplotypes) that spanned less than 150 kb and had an inter-marker distance shorter than than 50 kb were defined, leading to a compromise between the maximum number of SNPs and the minimum recombination probability within the block [14]. In total, 5756 SNP blocks were taken into account as multi-allelic markers and their haplotypes as alleles in the subsequent unbiased allelic diversity and heterozygosity analyses. Hereafter, SNP blocks are also referred to as multi-allelic markers.

### Genetic diversity

The following population genetic parameters were estimated: total number of alleles (*nA*), mean number of alleles per block (*mA*), allelic richness (*AR*), observed (*H*_*O*_) and expected heterozygosity (*H*_*E*_), number of private alleles (*npA*; alleles observed only in one population), frequency of private alleles (*fpA*) and number of common alleles (*ncA*, observed in all subpopulations) as described previously [13, 44, 45]. In addition, we used the number of semi-private alleles (*nspA*), defined as the alleles observed in two populations only, to further assess the allelic diversity and to consider any genetic and geographical closeness of the included breed pairs. Inbreeding coefficients of each animal *i* (*F*_*i*_) were estimated from the diagonal elements of the UAR matrix as *F*_*i*_= *UAR*_*(i,i)*_ − 1. To improve the presentation and discussion of the summary statistics related to diversity, we standardized and then plotted these statistics onto a map with a tessellated projection using the R-script available with the package *Tess* (http://membres-timc.imag.fr/Olivier.Francois/TESS_Plot.html).

To assess the subpopulation-differentiation, we used the *D*_*EST*_ estimator, which is analogous to the classical *G*_*ST*_ for multi-allelic loci but is unbiased and more suitable when the level of gene diversity is high [46]. In this approach, we used the dataset of the genotypes for 5756 multi-allelic SNP blocks in 115 breeds.

### Past effective population size based on linkage disequilibrium

We applied a linkage disequilibrium (LD) based method as implemented in the *SneP* tool [47] to estimate the historical and recent effective population size (*Ne*) for all the cattle breeds with sample size larger than 8. The estimation was performed on the SNPs with minimum and maximum distances equal to 20,000 and 10,000,000 bp, respectively, and by applying a recombination rate correction [48] and a sample size correction. The most recent effective population is represented by *Ne*_*5*_, (i.e. five generations ago), the effective population size in pre-industrial times (i.e. 50 generations or 250 years ago) is represented by *Ne*_*50*_, and in times close to domestication (10,000 years ago) by *Ne*_*2000*_. To improve the presentation and discussion of the effective population size across time and space, we standardized and then plotted *Ne*_*5*_, *Ne*_*50*_ and *Ne*_*2000*_ onto a map with a tessellated projection using the R-package *Tess* as described above.

### Phylogeny and population structure

We applied four phylogenetic and population structure analyses to infer relationships between animals and breeds. Two of these analyses rely on bi-allelic SNP genotypes and two on multi-allelic SNP-block genotypes. In addition, two of these analyses represent supervised clustering and two represent unsupervised clustering.

#### Supervised phylogeny of 115 cattle breeds

To elucidate the phylogenetic relationships among the studied populations, we implemented maximum likelihood (ML) methods using the TreeMix program [49]. In this approach, we used the dataset of SNPs genotyped in 115 breeds (Table 1), and set YAK as outgroup to root the tree. The second supervised approach used the allele frequencies of 5756 haplotype blocks to estimate the Nei’s unbiased *D*_*A*_-distances [50]. Then, the D_A_-distance matrix of 115 breeds was used to reconstruct the neighbor-net with the SplitsTree4 software and the neighbor-joining tree with the FigTree 1.4 software (http://tree.bio.ed.ac.uk/software/figtree/) [51]. We set YAK as outgroup to root the neighbor-joining tree.

**Table 1:** Parameters of genetic diversity in the 115 examined cattle breeds with 46,678 SNPs

Breed	*Nd*	*nA*	$\overline{nA}$	*mA*	$\overline{mA}$	*H*_*O*_	*H*_*E*_	*H*_*def*_	*npA*	$\overline{npA}$	*nspA*	$\overline{nspA}$	*fpA*	*F*	$\overline{F}$	*AR*	$\overline{AR}$	*H*_*o(SNPs)*_	*H*_*E(SNPs)*_	*Νe*_*5*_	$\overline{{Ne_{5} }}$	*Ne*_*50*_	*Ne*_*2000*_
Outgroups																							
YAK	26	9193		1.60		0.232	0.099	− 1.350	79		70		0.406	1.056		1.29		0.030	0.029	54		123	
GIR	24	24,111		4.19		0.486	0.464	− 0.049	78		139		0.041	0.647		2.63		0.169	0.169	48		304	3966
NDA	27	32,136		5.58		0.569	0.574	0.009	188		185		0.032	0.334		3.15		0.228	0.230	67		524	2737
Minor Asia																							
ATER	17	41,775		7.26		0.733	0.725	− 0.011	147		158		0.031	0.106		4.17		0.321	0.317	40		349	4338
ATBC	37	51,955		9.03		0.730	0.746	0.022	294		362		0.015	0.112		4.25		0.322	0.330	96		831	5040
ATSR	17	40,859		7.10		0.713	0.719	0.008	152		185		0.034	0.142		4.12		0.310	0.313	40		337	4274
ATSY	7	29,941		5.20		0.696	0.683	− 0.019	43		64		0.076	0.161		4.08		0.302	0.298	–		–	–
TRG	8	31,406	44,046	5.46	7.65	0.740	0.695	− 0.064	28	191.7	65	234.8	0.069	0.071	0.117	4.10	4.18	0.327	0.308	15	64	125	2952
Greece and Cyprus																							
CYP	12	27,328		4.75		0.629	0.627	− 0.004	32		55		0.115	0.261		3.41		0.271	0.271	18		119	2753
AGT	6	17,302		3.01		0.593	0.504	− 0.178	17		23		0.206	0.329		2.74		0.244	0.218	–		–	–
CRT	11	12,688		2.20		0.518	0.373	− 0.387	11		8		0.376	0.457		2.02		0.193	0.166	8		53	1753
NSY	7	19,634		3.41		0.608	0.551	− 0.102	11		16		0.188	0.242		2.97		0.268	0.249	–		–	–
GRB	41	49,079		8.53		0.701	0.742	0.056	142		208		0.021	0.108		4.18		0.314	0.334	85		618	4170
KAS	4	17,185		2.99		0.656	0.520	− 0.261	23		19		0.141	0.261		2.99		0.270	0.224	–		–	–
KEA	27	33,145		5.76		0.646	0.622	− 0.038	16		23		0.026	0.150		3.31		0.295	0.284	28		160	2791
PRG	9	26,666		4.63		0.704	0.643	− 0.094	13		31		0.073	0.096		3.58		0.319	0.293	15		109	2950
ROG	9	25,912		4.50		0.723	0.602	− 0.202	20		29		0.100	0.094		3.36		0.321	0.270	15		114	3099
SYK	17	37,037		6.43		0.664	0.698	0.049	67		71		0.050	0.149		3.91		0.300	0.316	29		210	3429
KTR	19	37,105	37,879	6.45	5.89	0.651	0.685	0.050	54	58.6	74	82.6	0.045	0.175	0.178	3.80	3.54	0.292	0.308	26	40	192	3441
South-east Europe																							
RHS	17	41,278		7.17		0.741	0.734	− 0.009	70		104		0.034	0.058		4.22		0.334	0.332	38		308	3905
MKB	22	45,364		7.88		0.721	0.744	0.031	106		156		0.025	0.076		4.27		0.326	0.337	51		425	3932
SRB	20	44,355		7.71		0.726	0.742	0.021	101		120		0.028	0.063		4.27		0.329	0.337	46		396	4099
PRE	29	44,126		7.67		0.752	0.733	− 0.025	73		92		0.025	0.028		4.12		0.343	0.335	58		420	3933
RMB	17	42,940		7.46		0.757	0.738	− 0.025	105		127		0.032	0.027		4.28		0.343	0.335	40		345	3825
SHB	17	39,748		6.91		0.727	0.724	− 0.004	84		104		0.039	0.062		4.13		0.331	0.329	34		275	3664
DGB	21	36,322		6.31		0.729	0.700	− 0.041	30		41		0.052	0.053		3.86		0.335	0.322	31		228	3398
DBB	25	37,724		6.55		0.725	0.703	− 0.031	34		41		0.038	0.060		3.87		0.334	0.324	34		257	3298
MAB	43	51,066		8.87		0.738	0.745	0.010	148		180		0.013	0.042		4.20		0.339	0.342	97		712	3748
LKB	27	44,381		7.71		0.743	0.729	− 0.020	85		105		0.024	0.034		4.10		0.340	0.333	54		406	3709
SKB	14	35,556		6.18		0.717	0.707	− 0.013	33		48		0.048	0.070		4.00		0.328	0.325	25		205	3312
MNB	19	41,711		7.25		0.731	0.730	− 0.001	65		81		0.032	0.045		4.16		0.336	0.335	41		337	3679
BHB	18	30,993		5.38		0.687	0.642	− 0.070	21		44		0.064	0.113		3.47		0.311	0.291	23		154	3161
HRB	28	48,122	42,800	8.36	7.44	0.745	0.745	0.001	106	82.2	147	106.5	0.020	0.027	0.051	4.26	4.10	0.340	0.341	68	51	583	3916
East Podolian																							
HRI	28	40,285		7.00		0.709	0.704	− 0.007	25		58		0.039	0.074		3.88		0.323	0.322	49		322	3414
HRP	24	29,711		5.16		0.679	0.639	− 0.063	16		31		0.092	0.129		3.38		0.306	0.289	26		149	2959
UKP	21	31,176	34,188	5.42	5.94	0.689	0.656	− 0.051	27	22.6	38	43.4	0.053	0.109	0.103	3.50	3.61	0.313	0.298	29	36	161	2952
Tyrrhenian																							
PODO	25	43,858		7.62		0.715	0.731	0.022	75		95		0.023	0.074		4.13		0.325	0.332	56		434	3756
CINI	30	43,242		7.51		0.715	0.727	0.016	44		77		0.025	0.075		4.06		0.325	0.331	60		434	3882
MOSI	29	37,742		6.56		0.690	0.688	− 0.002	26		46		0.021	0.112		3.75		0.312	0.311	51		300	3546
RSIC	24	40,468		7.03		0.739	0.719	− 0.028	21		41		0.024	0.041		4.02		0.337	0.329	45		342	3632
MOSA	28	39,565		6.87		0.719	0.707	− 0.017	22		39		0.037	0.068		3.91		0.325	0.320	53		349	3816
SARD	30	44,722		7.77		0.710	0.726	0.022	76		90		0.021	0.059		4.08		0.326	0.333	66		533	3633
SBRU	10	32,211		5.60		0.697	0.689	− 0.012	15		18		0.050	0.080		3.95		0.319	0.317	21		173	3226
CORS	30	44,708		7.77		0.722	0.724	0.002	120		119		0.022	0.050		4.06		0.331	0.330	67		535	3572
AGER	22	34,676		6.02		0.705	0.688	− 0.025	2		15		0.023	0.072		3.76		0.327	0.320	32		222	2966
MARE	34	37,470		6.51		0.695	0.690	− 0.006	44		47		0.028	0.105		3.74		0.313	0.312	53		294	3367
CHI	12	32,476		5.64		0.702	0.677	− 0.037	21		42		0.042	0.093		3.81		0.320	0.310	24		166	2928
MPIS	15	21,614		3.76		0.639	0.539	− 0.185	7		8		0.152	0.173		2.82		0.286	0.246	15		86	2203
CALV	24	28,886		5.02		0.640	0.617	− 0.036	4		19		0.057	0.172		3.24		0.288	0.280	29		146	2841
MCH	21	37,548		6.52		0.711	0.696	− 0.021	39		50		0.040	0.077		3.86		0.320	0.314	45		282	3311
RMG	18	34,890		6.06		0.677	0.667	− 0.014	158		153		0.032	0.127		3.69		0.307	0.300	34		191	3245
GARF	23	26,502		4.60		0.645	0.618	− 0.044	13		16		0.077	0.156		3.21		0.294	0.283	22		121	2833
PONT	13	25,804		4.48		0.631	0.599	− 0.055	3		18		0.103	0.170		3.20		0.287	0.274	17		104	2466
MODE	23	35,203		6.12		0.705	0.687	− 0.026	27		31		0.045	0.069		3.75		0.324	0.315	35		239	3111
CABA	22	36,019		6.26		0.715	0.693	− 0.031	21		22		0.027	0.052		3.81		0.329	0.319	38		258	3132
REGG	26	38,053		6.61		0.724	0.699	− 0.036	52		56		0.046	0.049		3.83		0.333	0.322	45		293	3503
PMT	16	39,962		6.94		0.737	0.719	− 0.026	171		171		0.033	0.029		4.12		0.339	0.327	36		294	3284
BURL	24	36,125	37,025	6.28	6.43	0.713	0.696	− 0.023	11	45.1	19	55.7	0.047	0.077	0.087	3.81	3.79	0.335	0.327	40	44	267	3014
Alpine																							
PRDO	23	33,445		5.81		0.685	0.673	− 0.017	15		17		0.038	0.088		3.64		0.314	0.309	38		250	2912
OVAR	31	39,534		6.87		0.731	0.706	− 0.035	30		29		0.035	0.038		3.87		0.335	0.325	48		330	3409
REND	24	33,939		5.90		0.687	0.671	− 0.024	11		23		0.053	0.092		3.63		0.314	0.308	44		233	3181
BPUS	24	38,621		6.71		0.717	0.703	− 0.021	25		31		0.032	0.052		3.88		0.330	0.324	42		321	3335
PUST	24	31,615		5.49		0.701	0.667	− 0.052	16		17		0.059	0.075		3.57		0.321	0.306	32		197	3075
SIC	26	39,920		6.94		0.732	0.714	− 0.025	28		39		0.032	0.035		3.97		0.336	0.328	49		355	3562
PIN	29	38,594		6.71		0.714	0.701	− 0.020	27		39		0.024	0.066		3.84		0.332	0.326	50		309	3408
TGV	50	38,939		6.76		0.700	0.690	− 0.014	36		45		0.023	0.071		3.72		0.322	0.318	67		344	3187
MWF	46	35,855		6.23		0.710	0.675	− 0.053	21		25		0.039	0.058		3.58		0.327	0.311	45		252	3128
OBV	35	36,925		6.42		0.695	0.682	− 0.020	17		26		0.028	0.074		3.68		0.319	0.313	66		333	3390
BBV	50	35,287		6.13		0.656	0.643	− 0.020	10		15		0.010	0.100		3.39		0.301	0.296	65		233	2797
DFV	50	40,079		6.96		0.692	0.684	− 0.013	26		41		0.012	0.075		3.69		0.320	0.316	98		445	3123
FGV	50	37,494		6.51		0.704	0.682	− 0.031	15		36		0.032	0.067		3.65		0.325	0.316	64		311	3148
VOG	18	34,749		6.04		0.721	0.688	− 0.048	12		22		0.039	0.045		3.80		0.331	0.317	33		245	3128
ABO	22	33,385		5.80		0.706	0.666	− 0.061	21		18		0.040	0.054		3.60		0.325	0.306	37		240	3005
MON	28	31,738		5.51		0.691	0.647	− 0.068	11		15		0.065	0.083		3.43		0.318	0.298	36		209	3003
TAR	37	37,339	36,709	6.49	6.38	0.692	0.680	− 0.018	18	20.5	47	30.0	0.022	0.079	0.070	3.66	3.67	0.318	0.314	65	56	342	3228
France																							
R DBI	29	26,163		4.55		0.606	0.578	− 0.048	25		34		0.174	0.200		2,99		0.277	0.265	28		127	2615
SAL	26	36,384		6.32		0.668	0.667	0.000	26		40		0.043	0.117		3.62		0.305	0.305	58		321	2942
AUB	22	37,489		6.51		0.691	0.685	− 0.008	32		49		0.028	0.079		3.79		0.317	0.314	51		349	2840
LIM	48	42,891		7.45		0.708	0.705	− 0.003	65		75		0.015	0.066		3.85		0.326	0.325	106		563	3274
CHR	39	43,444		7.55		0.720	0.719	− 0.001	67		63		0.018	0.061		3.97		0.335	0.335	88		482	3340
PAR	17	36,898		6.41		0.724	0.701	− 0.033	52		53		0.035	0.044		3.93		0.333	0.322	34		262	3242
BAQ	33	40,280		7.00		0.706	0.700	− 0.009	53		73		0.021	0.065		3.83		0.325	0.322	69		426	3291
GAS	22	37,288	38,387	6.48	6.67	0.699	0.687	− 0.018	49	48.9	65	58.9	0.028	0.071	0.087	3.80	3.73	0.320	0.314	49	67	317	3071
Iberian																							
MNRQ	30	26,609		4.62		0.637	0.605	− 0.054	28		27		0.173	0.166		3.12		0.292	0.277	29		128	2868
MALL	30	20,486		3.56		0.556	0.521	− 0.067	14		26		0.211	0.276		2.65		0.247	0.236	19		81	2456
NGAN	14	35,429		6.16		0.660	0.702	0.059	56		67		0.053	0.143		3.96		0.301	0.320	28		221	3208
CANA	30	31,527		5.48		0.645	0.614	− 0.051	16		36		0.151	0.158		3.24		0.292	0.279	31		168	2801
MARI	22	30,016		5.21		0.656	0.622	− 0.055	22		42		0.116	0.147		3.30		0.299	0.284	27		148	2686
ALEN	10	29,018		5.04		0.654	0.655	0.001	98		86		0.080	0.166		3.67		0.297	0.294	19		136	3104
BAR	14	32,044		5.57		0.659	0.663	0.006	29		54		0.051	0.145		3.68		0.301	0.302	31		199	2738
MARO	19	35,039		6.09		0.687	0.677	− 0.014	40		65		0.037	0.108		3.73		0.313	0.309	45		265	3147
SYG	11	30,662	29,205	5.33	5.07	0.670	0.667	− 0.004	43	31.3	35	43.2	0.063	0.129	0.169	3.75	3.32	0.305	0.303	20	28	149	2802
North-west Europe																							
BPN	15	33,384		5.80		0.726	0.689	− 0.054	28		46		0.055	0.047		3.83		0.336	0.319	26		200	3033
NOR	30	34,001		5.91		0.698	0.664	− 0.052	38		61		0.055	0.091		3.55		0.322	0.307	43		234	3072
MAN	20	34,940		6.07		0.683	0.664	− 0.029	23		22		0.032	0.123		3.64		0.320	0.312	44		244	2941
BBB	45	39,395		6.84		0.698	0.690	− 0.010	40		46		0.028	0.103		3.72		0.328	0.325	67		329	3110
LKF	22	31,859		5.53		0.678	0.658	− 0.030	10		25		0.057	0.129		3.54		0.316	0.307	34		187	2810
HF	50	37,888		6.58		0.696	0.685	− 0.016	14		29		0.011	0.127		3.65		0.334	0.329	72		279	2877
GNS	16	28,903		5.02		0.634	0.623	− 0.016	89		65		0.039	0.175		3.36		0.292	0.286	29		159	2606
JSY	49	31,507		5.47		0.624	0.619	− 0.007	20		28		0.020	0.185		3.23		0.287	0.286	74		223	2641
HER	41	36,659		6.37		0.653	0.676	0.034	55		64		0.028	0.229		3.57		0.320	0.332	79		227	2286
SHR	13	25,626		4.45		0.566	0.575	0.016	4		15		0.039	0.300		3.12		0.267	0.274	27		128	2131
KRY	14	27,681		4.81		0.678	0.644	− 0.053	57		67		0.083	0.144		3.46		0.317	0.300	22		131	2698
DXT	16	31,660		5.50		0.625	0.667	0.062	32		26		0.065	0.202		3.64		0.292	0.312	31		185	2841
GLW	40	34,358		5.97		0.635	0.652	0.025	24		36		0.047	0.186		3.45		0.296	0.304	82		275	2744
AAN	48	35,756		6.21		0.659	0.668	0.014	30		42		0.026	0.188		3.53		0.317	0.322	83		249	2617
HGL	27	28,798		5.00		0.606	0.605	− 0.001	33		47		0.045	0.231		3.18		0.281	0.282	57		181	2524
NRC	34	36,853		6.40		0.702	0.686	− 0.023	20		39		0.033	0.101		3.70		0.333	0.325	57		268	3016
SERC	24	35,396		6.15		0.690	0.678	− 0.018	32		35		0.069	0.113		3.68		0.325	0.320	47		239	2839
FJL	22	35,464		6.16		0.700	0.680	− 0.030	67		74		0.083	0.093		3.72		0.319	0.310	42		242	2946
FIAY	42	37,043		6.44		0.686	0.677	− 0.014	19		46		0.018	0.111		3.61		0.323	0.318	65		281	2942
FINE	20	38,958		6.77		0.692	0.711	0.026	78		100		0.048	0.097		3.99		0.317	0.324	41		321	3608
FINW	35	38,966		6.77		0.696	0.693	− 0.004	59		81		0.040	0.095		3.77		0.317	0.316	56		316	3372
FINN	18	32,166		5.59		0.719	0.670	− 0.073	27		42		0.067	0.069		3.64		0.327	0.305	27		190	3140
YARO	20	36,065	34,908	6.27	6.06	0.707	0.686	− 0.029	58	34.9	69	47.0	0.068	0.080	0.142	3.78	3.58	0.324	0.315	39	57	246	3265

*Nd*, number of genotyped animals; *nA* and $\overline{nA}$, total and mean number of observed alleles within subpopulation; *mA*, nA/5756 (number of blocks); *H*_*O*_, average observed heterozygosity within subpopulation; *H*_*E*_, average expected heterozygosity within subpopulation; *npA* and $\overline{npA}$, number and mean number of private alleles; n*spA* and $\overline{nspA}$, number and mean number of alleles present only in two subpopulations; *f*pA, average frequency of private alleles, F and $\overline{F}$, inbreeding coefficient per breed and per group; *AR* and $\overline{AR}$, number and mean value of allelic richness; *Ho*_(SNP)_ and *H*_*E*(SNP)_, observed and expected heterozygosity within subpopulation by SNPs; Ne_5_ and $\overline{{Ne_{5} }}$, effective population number for five generations back per breed and per group; Ne_50_ and Ne_2000_, effective population number for fifty and two thousand generations back

#### Unsupervised population structure analyses

Similar to the above analysis, first we examined the population structure based on SNP genotypes. For this purpose, we conducted clustering with the program Admixture 1.23 [52] under the assumption that the number of clusters is equal to *K*, with *K* ranging from 1 to 115, i.e. the number of breeds plus 1. Since the admixture analysis does not need an outgroup, we used the dataset without YAK. To assess the quality of clustering and thus infer the most likely *K,* we performed 10 cross-validations [53] and estimated the cross-validation error for each *K*. To illustrate the results of the admixture analyses, we used the Python package Pong [54]. The second unsupervised approach used the proportion of genome-wide shared SNP-block alleles (*PS*) among all pairs of 2858 animals. The PS matrix was then converted into an allele sharing distance matrix (D_PS_) as described by Bowcock et al. [55]. Multidimensional scaling (MDS) [56] was used to project the multidimensional D_PS_ distance matrix onto a two-dimensional (2D) plane for the 115 breeds. For a better illustration and interpretation of the MDS results, for each breed, we calculated the mean MDS-coordinates of all the individuals, which correspond to the center of each breed symbol (circle), and then we estimated the standard deviation (SD) around that center. Specifically, for each breed, we calculated the spatial distance of each individual to the group center by applying the Pythagorean theorem, assuming that the hypotenuse is the distance between the center of the breed symbol and the position per individual. Then, we estimated the SD of these spatial distances. Finally, the SD was used as the radius around the breed center symbol, as a proxy for spatial dispersion of animals of each breed. For visualization purposes, plot dimensions were proportionally adjusted in R, considering a 1-inch (= 0.254 cm) length as the longest radius.

We calculated the mean *D*_*PS*_ within breed to show the level of breed differentiation. Mean *D*_*PS*_ values were standardized and then plotted as a tessellated projection onto a map, using the R-package *Tess* as described above.

### D-statistics analysis

To investigate the historical admixture between *taurine* and *indicine* cattle, the D-statistics [57] was computed by using the qpDstats tool of the AdmixTool software package [58]. In brief, for a set of three populations P1, P2 and P3, and an outgroup O that fits to this phylogeny: (((P1,P2),P3).O), the numbers of shared alleles between P1 and P3 (*BABA*) and, P2 and P3 (*ABBA*) are calculated by assuming that allele “*A*” represents the ancestral allele and allele “*B*” the derived allele. The significant excess of either “*ABBA*” or “*BABA*” indicates admixture between populations P2 and P3, or P1 and P3, respectively. We used YAK as outgroup O and *Bos indicus* (GIR) as P3. We selected Highland cattle that originate from the most northwestern part of Europe (Scotland) as P2 and, thus as a *Bos taurus* breed with the lowest, if any, admixture level with *Bos indicus*. All other cattle breeds were tested as P1. To present the gradient of *Bos indicus* genes in European taurine cattle, we standardized and then plotted the D-values onto a map with a tessellated projection using the R-package *Tess* as described above. The D-values with Z > |3| were considered as significant and indicated on the map.

## Results

### Genetic diversity

**Fig. 2:**
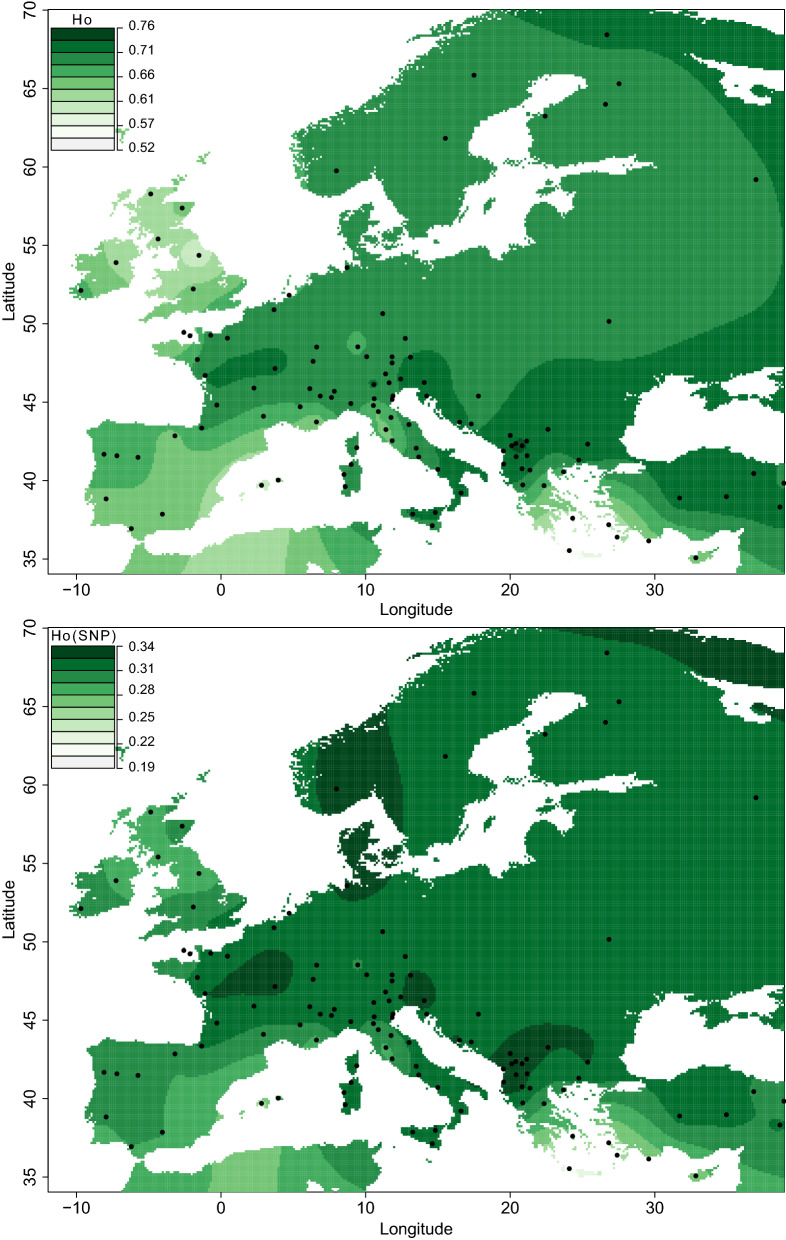

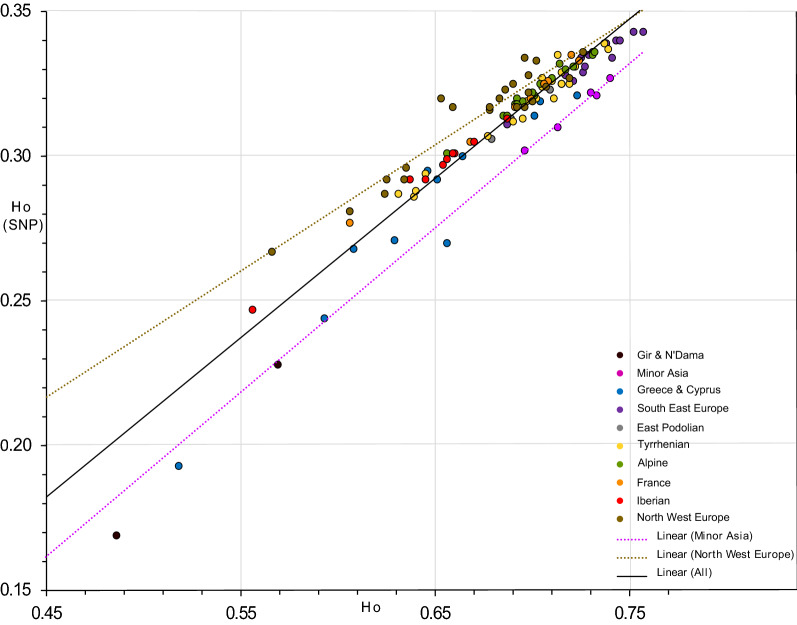
Tessellated projection and value distribution plot of observed heterozygosity estimated based on multi-allelic SNP-blocks (*H*_*O*_) and bi-allelic SNPs (*H*_*O[SNP]*_)

In total, 590 common alleles were detected among the 115 breeds studied, which represents only 0.7% of the total number (80,720) of alleles. All estimators of genetic diversity for the breeds studied here were highly differentiated among the predefined geographical groups (Table 1**)**. The geographic group of Minor Asia displays the highest average value for almost all the estimators of allelic diversity used, i.e. for: total number of alleles (*nA*), number of alleles per haplotype block (*mA*), number of private (*npA*), number of semi-private alleles (*nspA*) and allelic richness (*AR*) (Table 1**)**. The heterozygosity estimates based on multi-allelic SNP blocks and on bi-allelic SNPs are highest for the South East European Buša breeds. The only diversity parameter, which indicates a higher diversity in the central Europe group than in the Minor Asian group, is the observed heterozygosity estimator based on bi-allelic SNPs (*H*_*O[SNP]*_) (Table 1 and Fig. 2). Intermediate values were found for the breeds in the Greek and Cyprus group for mean number of observed alleles ($\overline{nA}$ = 37,879), mean number of private ($\overline{npA}$ = 58.6) and semi-private alleles ($\overline{nspA}$ = 82.6). The Greek and Cyprus group showed the highest mean inbreeding coefficient ($\overline{F}$ = 0.178) and relatively low values for: mean allelic richness ($\overline{AR}$ = 3.54), mean observed ($\overline{{H_{O} }}$ = 0.656) and mean expected heterozygosity ($\overline{{H_{E} }}$ = 0.641). Within the Greek and Cyprus cattle breeds, CRT exhibited the lowest values for all genetic diversity parameters and, at the same time, the highest frequency of private alleles (*fpA* = 0.376) as well as the highest average inbreeding coefficient (*F* = 0.457). For GRB, most of the diversity estimates had the highest values but the frequency of private alleles (*fpA* = 0.021) and the inbreeding coefficient (*F* = 0.108) were low. Generally, all the analyzed island populations except the KEA breed, which was sampled on the island of Kea and on mainland, had very high levels of inbreeding and very low diversity parameters (Table 1). GRB and the South East Europe Buša cattle shared similar values for almost all diversity parameters. The tessellated projection of diversity statistics provided strong support for a high allelic diversity in breeds from Anatolia and part of South East Europe. Based on Additional file 3: Figure S1, a southeast to northwest gradient of genetic diversity could be inferred, but it is interrupted by the genetic diversity parameters of the Greek island breeds. However, if the Greek island breeds are excluded, this possible southeast to northwest gradient of genetic diversity remains consistent (see Additional file 3 Figure S1). To illustrate a possible ascertainment bias of the SNP chip data, we present the tessellated projection of the observed heterozygosity estimations based on multi-allelic blocks (*H*_*O*_) and bi-allelic SNPs (*H*_*O[SNP]*_) side-by-side in Fig. 2. This shows that *H*_*O[SNP]*_ suggests a high level of genetic diversity in some Alpine and North West European breeds, whereas *H*_*O*_ highlights breeds from South East Europe and Anatolia as having the highest level of diversity. The ascertainment bias of SNP chip data was further highlighted by the scatterplot of *H*_*O[SNP]*_ versus *H*_*O*_ in Fig. 2. Both *H*_*O[SNP]*_ and *H*_*O*_ are estimators of the true diversity. Therefore, the diversity of the breeds placed above the overall trend line (e.g. North West Europe) is overestimated by *H*_*O[SNP]*_ and the diversity of the breeds placed below this line (e.g. Minor Asia) is underestimated by *H*_*O[SNP]*_.

### Effective population size *Ne*

The weighted mean of the current effective population size (*Ne*_*5*_) is relatively small and ranges from 28 in the Iberian to 67 in the French geographic group. The Greek and Cyprus group showed a small average value (*Ne*_*5*_ = 40). Only GRB showed a larger *Ne*_*5*_*(Ne*_*5*_ =* 85)* than the weighted average of the group (Table 1). Going back in the past, the effective population size increased faster in the Buša group than in other European cattle groups. Consequently, during the pre-industrial time (*Ne*_*50*_ 250 years ago), the effective population size was clearly larger for the South East Europe Buša (*Ne*_*50*_ = 399) group than for other European breed groups. Only the Minor Asian group showed a larger value (*Ne*_*50*_ = 549) during pre-industrial times. A comparable trend was also observed for effective population size 10,000 years ago (*Ne*_*2000*_). The values for the GRB and Buša groups were comparable and differed from those for the other Greek cattle breeds. The 11 indigenous CRT animals sampled on the island of Crete showed the highest inbreeding level and the smallest effective population size (*Ne*_*5*_, *Ne*_*50*_ and *Ne*_*2000*_) in the entire dataset (Table 1) and see Additional file 4: Figure S2. The tessellated projections provide strong support to the observation that as we go back in time the effective population size increases in regions closer to the domestication center and decreases in North West Europe (see Additional file 4: Figure S2). As *Ne* is inversely correlated to the extent of LD, our results suggest that the level of LD is high in the fragmented breeds under extinction pressure, which is most probably caused by uncontrolled inbreeding.

### Genetic differentiation

Pairwise population differentiation values as estimated by the unbiased estimator developed for multi-allelic markers, *D*_*EST*_, are in Additional file 5: Table S4). Τhe *D*_*EST*_ values between 112 cattle breeds (YAK, GIR and NDA excluded) ranged from 0.001 to 0.462. The breeds and breed groups with high allelic diversity and high heterozygosity showed a very low level of differentiation. For example, five breeds of the Minor Asia group showed an average differentiation to each other of only 0.006 and the Buša breeds a value of 0.050. On the other hand, the Greek and Cyprus breeds were highly differentiated ($\overline{{D_{EST} }}$ = 0.250), followed by the Iberian group ($\overline{{D_{EST} }}$ = 0.189) and the East Podolian group ($\overline{{D_{EST} }}$ = 0.180) see Additional file 5 Table S4). Similar trends were also observed when breeds from one pre-defined group were compared to all the other breeds, i.e. the level of differentiation was lowest for the Buša breeds (0.113) and highest for the Greek and Cyprus breeds (0.224).

Within the Greek and Cyprus group, low pairwise *D*_*EST*_ values were obtained between two mainland breeds i.e. GRB-SYK (= 0.073) and high values were obtained between two island breeds i.e. CRT-AGT (*D*_*EST*_ = 0.413). It is remarkable that the CRT breed showed the highest differentiation level among all the investigated breeds ($\overline{{D_{EST} }}$ = 0.391). Again, the GRB and Buša breeds shared comparable *D*_*EST*_ values.

In addition, we used average allele sharing distance (D_PS_) between animals within a breed as a measure of the breed-level differentiation. As shown in Additional file 6: Figure S3), the highest D_PS_ values were observed for most of the Buša and Anatolian breeds together with the Greek GRB and the Italian PODO breeds, whereas the lowest values were observed for the Greek island breeds.

### Genetic distances and phylogenetic neighbor net

**Fig. 3:**
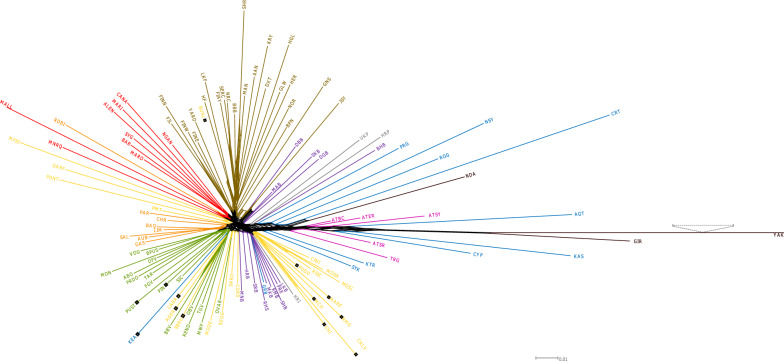
Neighbor-network based on pairwise Nei’s D_A_ genetic distances among 115 breeds. Special square marks represent the influence of East-Podolian (grey), Alpine (green) and North-West (olive green) groups. Dotted lines indicate the shortened branch length of Yak to improve visibility

**Fig. 4:**
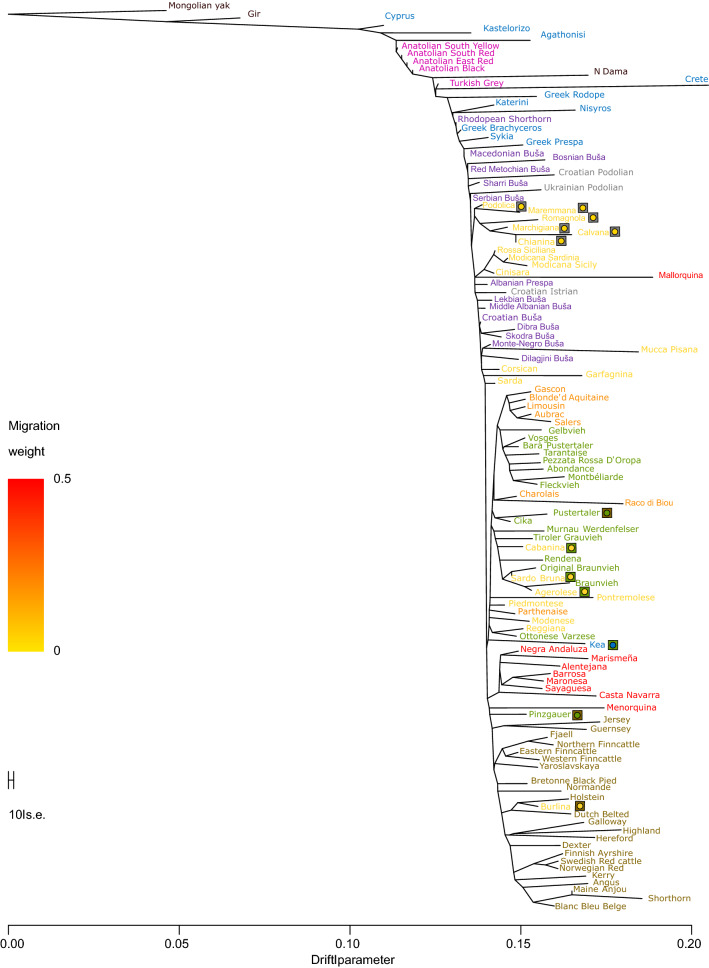
Maximum likelihood (ML) tree inferred from genome-wide allele frequency data by methods implemented in the *T*_*REEMIX*_ program. The ML dendrogram of the relationships between the examined cattle populations was rooted with the Mongolian yak as an outgroup breed

We estimated Nei’s genetic distance *D*_*A*_ based on multi-allelic SNP blocks and present the values as a neighbor-net (Fig. 3) and as a neighbor-joining tree routed by YAK (see Additional file 7 Figure S4). In Fig. 3 to improve the visibility of the main part of the tree, we shortened the branch length for YAK. Among the Greek and Cyprus group, the island breeds CYP, AGT and KAS were placed close to *Bos indicus*. This is also the case for the Anatolian breeds, which form a cluster with the aforementioned breeds. Interestingly, the GRB breed from the mainland Greek group, is positioned within the cluster of the Buša breeds with short branches. On the opposite, for the island breeds CRT, AGT, KAS and NSY, long branches result from a high inbreeding level. KTR and SYK, which represent Greek podolian cattle, are placed between the TRG and Italian podolic breeds. KEA is the only Greek breed that clusters together with some breeds from the Alpine region, as a result of crossbreeding that occurred between indigenous Greek cattle and some Alpine cattle breeds. This cluster includes also some Italian breeds with Brown-Swiss influence (CABA, AGER and SBRU). In addition, we used the allele counts of bi-allelic SNPs and reconstructed the phylogeny with the TreeMix program (Fig. 4) by using YAK to root the tree. Compared to other European cattle breeds, CYP, KAS and AGT were closer to the root of the tree and thus, closer to GIR, the *Bos indicus* representative. The aforementioned Greek breeds are gradually followed by the cattle breeds from Minor Asia, Greece, Bulgaria, North Macedonia, Kosovo and Serbia. In agreement with the neighbor-net, KEA is the only Greek breed that clusters in the Alpine cluster.

The remaining Buša breeds sampled along the Ionian-Adriatic route, i.e. Albania, Montenegro and Dalmatia, were placed after two clusters of Italian breeds. Our phylogenetic analyses, both with multi-allelic (Fig. 3) and bi-allelic markers (Fig. 4), do not aggregate the so-called Podolian or gray steppe cattle breeds (TRG, KTR, SYK, HRI, HRP, and UKP) in a single separate cluster, instead they are scattered along the phylogenetic tree and are positioned closer to their geographic neighbor than to the hypothetical steppe cattle or Podolian group. On the one hand, TRG, KTR, and SYK are positioned between the Anatolian and some of the Greek and North Macedonian breeds. On the other hand, HRI, HRP and UKP do not form an own cluster but are placed among some of the Buša neighbor breeds (Fig. 4). The Italian-podolian breeds form a separate cluster, which is placed between some of the Buša and other Italian breeds and the East podolian breeds. Finally, in the phylogenetic tree reconstructed based on SNP allele counts (TreeMix), for some samples with a high inbreeding level (e.g. CRT and BHB) long branches are observed whereas for less differentiated and highly diverse breeds (e.g. TRG and RMB) short or no branches are found.

### Assessment of population structure using unsupervised heuristic and unsupervised model-based methods

**Fig. 5:**
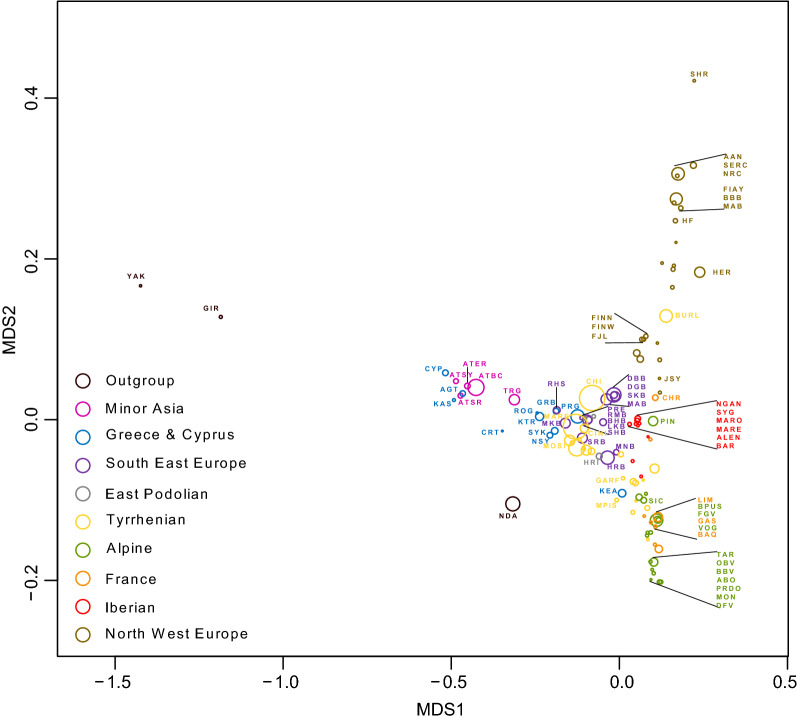
MDS projection of the estimated allele sharing distance matrix (D_PS_) among 115 breeds using multi-allelic SNP blocks. The position of each breed is represented with a circle in which the centre is the average position of all animals of the breed with a radius equal to the SD (standard deviation)

We used multi-allelic SNP blocks to estimate the allele sharing distance matrix (D_PS_) among 2858 animals and projected these by multidimensional scaling (MDS) on a two-dimensional (2D) plane. The MDS projection of 115 breeds is shown in Fig. 5. Αlong the first dimension of MDS (MDS1), we observe that the Anatolian, Greek and Cyprus breeds have an intermediate position between *Bos indicus* and the remaining European cattle breeds. CYP, AGT and KAS cluster together with the Anatolian breeds, except TRG. Subsequently, the mainland Greek breeds (SYK, KTR, PRG, ROG, and GRB) and the Nisyros island breed (NSY) cluster in the geographic region corresponding to some of the Buša breeds (RHS, MKB, PRE, RMD, SHD, and BHB; South East geographic group), some of the breeds from the East Podolian geographic group (UKP, and HRP) and some Italian breeds (Tyrrhenian group) of South and Central Italy (SINI, MOSA, MOSI, and RSIC) including all Italian podolic breeds (PODO, MARE, RMG, CALV, CHI, and MCH). CRT is isolated from the other two main Greek breed clusters. KEA is positioned in the geographic region of some Alpine and Italian breeds, showing a closer relationship to these breeds than to its own (Greek) geographic cluster. The second dimension of MDS (MDS2) separates the breeds of North Europe and Alpine geographic area from the breeds of central, west and southern Europe.

**Fig. 6:**
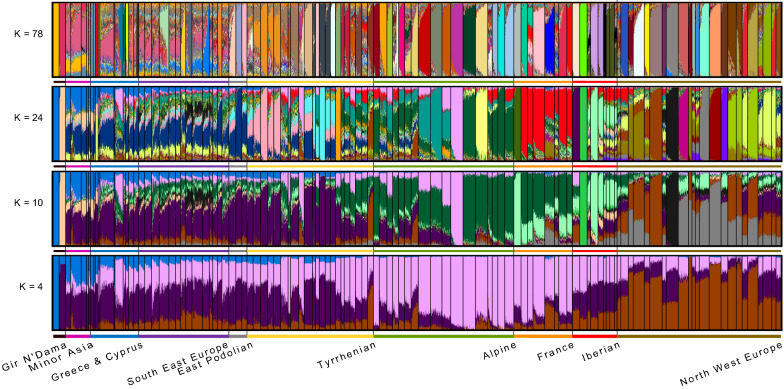
Model-based clustering of the estimated membership fractions of individuals from all examined breeds. Each cluster is indicated by a different color and each individual is shown as a vertical bar, at *K *= 4, *K *= 10, *K *= 24 and *K *= 78. The *K*-value of 78 represents the lowest cross-validation error (*cv *= 0.5358). The heights of the colored segments are proportional to genotype memberships. The names of the breed groups are given according to their geographical distributions as shown in Fig. 1. For a full definition of the breed groups (see Additional file 1: Table S1). From left to right within each group are presented the following breeds: Minor Asia: ATER, ATBC, ATSR, ATSY, TRG; Greece and Cyprus: CYP, AGT, CRT, NSY, GRB, KAS, KEA, PRG, ROG, SYK, KTR; South East Europe: RHS, MKB, SRB, PRE, RMB, SHB, DGB, DBB, MAB, LKB, SKB, MNB, BHB, HRB; East Podolian: HRI, HRP, UKP; Tyrrhenian: PODO, CINI, MOSI, RSIC, MOSA, SARD, SBRU, CORS, AGER, MARE, CHI, MPIS, CALV, MCH, RMG, GARF, PONT, MODE, CABA, REGG, PMT, BURL; Alpine: PRDO, OVAR, REND, BPUS, PUST, SIC, PIN, TGV, MWF, OBV, BBV, DFV, FGV, VOG, ABO, MON, TAR; France: RDBI, SAL, AUB, LIM, CHR, PAR, BAQ, GAS; Iberian: MNRQ, MALL, NGAN, CANA, MARI, ALEN, BAR, MARO, SYG; North West Europe: BPN, NOR, MAN, BBB, LKF, HF, GNS, JSY, HER, SHR, KRY, DXT, GLW, AAN, HGL, NRC, SERC, FJL, FIAY, FINE, FINW, FINN, YARO

The Admixture analysis presents the second unsupervised clustering method applied to 2832 animals (26 Mongolian yak excluded) using the genotypes of bi-allelic SNPs. The lowest cross-validation error (*cv error* = 0.5358) was determined at *K* = 78. Figure 6 presents the clustering at *K* = 4, 10, 24 and 78. The reasons for choosing *K* = 78 are that: (i) with a *K* larger than 4, animals from highly selected breeds start to separate into distinct clusters; (ii) the clusters at *K* = 10 represent the number of pre-defined geographical breed groups plus 1, i.e. nine groups of European *Bos taurus* plus NDA and GIR; (iii) the cross-validation error decreases almost linearly from *K* = 1 to *K* = 24; and (iv) the clustering with the lowest cross-validation error is at *K* = 78. Additional file 8: Figure S5 presents the Admixture results with the default color assignment performed by the Pong package for *K* = 2 till 26. All breeds from Minor Asia and most of the South East geographic groups, as well as GRB, showed a high level of complex admixture even at *K* = *78*. All highly selected breeds and isolated breeds formed their own cluster. GRB and KTR, and SYK to a lesser degree, shared the same pattern with most of the breeds belonging to the South East group with evidence of shared ancestry between these and the Anatolian breeds. The highly inbred and differentiated island breeds assigned to separate clusters. The same was also observed for small-sized mainland populations, such as PRG and ROG. At *K* = 24, a significant proportion of the Alpine ancestry (OBV, and BBV) was estimated for KEA, which is also retained at the most probable true *K* = 78.

### D-statistics analysis

**Fig. 7:**
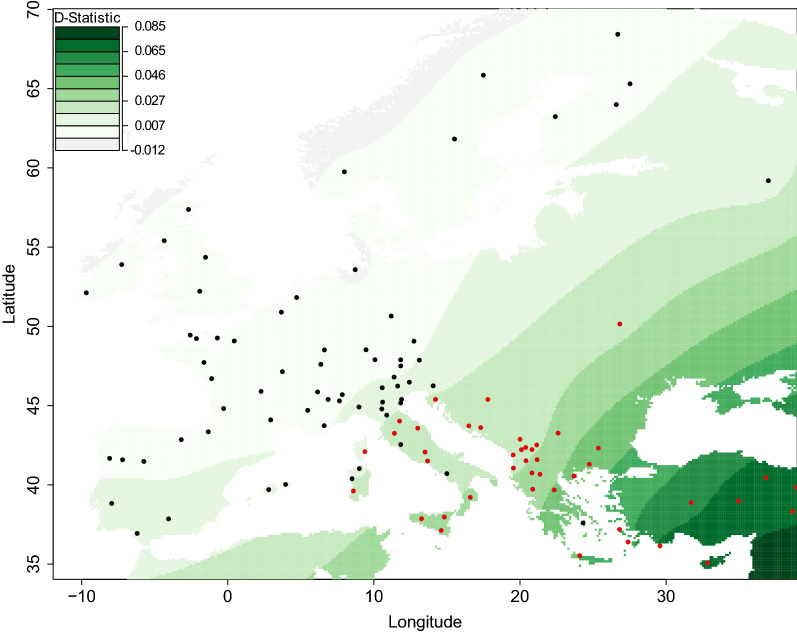
Tessellated projection of D-statistics values. Red dots represent a significant influence of *Bos indicus* (Z > |3|)

The historical admixture between taurine and indicine cattle was confirmed for most of the cattle breeds that originate from Anatolia, Cyprus, Greek and South East Europe and most of the Central and South Tyrrhenian breeds. The KEA, AGER and SBRU breeds, which are influenced by the Alpine group, as well as the MPIS and SARD breeds deviate from the above described general geographic trend. In Fig. 7, the values of all these significant D-statistics (Z < |3|) are shown with a red spot on a tessellated map to highlight the gradient of the *Bos indicus* introgression from the Southeast to the Northwest direction. The results provide strong evidence that support the influence of *Bos indicus* along the Balkan continental route and along the Mediterranean route up to North Italy.

## Discussion

In this study, we focused on the genome-wide structure of 11 indigenous cattle populations, 10 from Greece and one from Cyprus. The dataset included all the indigenous cattle populations that are reared in Greece and Cyprus and considered to be under constant risk mainly since the 1970s and onwards. An essential reason for this situation is the increased use of sires from the so-called cosmopolitan breeds mostly from the mainland. Sampling included as many individuals as possible from the whole Greek territory (Fig. 1). Some island indigenous breeds (CYP, AGT, KAS, and NSY) were represented by very small sample numbers (Table 1). However, due to the aforementioned limited population sizes and limitations regarding the herds (i.e. highly related, almost feral and difficult to handle), it was not realistic to consider that the number of samples can be increased. For the remaining Greek breeds, a sufficient number of 10 or more of the most representative individuals of the local breeds were sampled. Therefore, this study represents the most complete and up-to-date dataset available for the indigenous Greek cattle and integrated into the most complete Minor Asia and European sample collection.

Improving our knowledge of the genetic diversity within and among local breeds is an issue of crucial importance for implementing further conservation programs that are necessary for sustainable development and future animal farming in changing environments [34]. This could be particularly important for traditional unselected breeds that cover a geographical area close to the domestication center [32].

In spite of the ascertainment bias of the BovineSNP50 chip data [13, 43], it was highly informative for the analyzed Greek and Cyprus cattle populations as reflected by the consistent values obtained for various parameters of genetic diversity levels. These parameters indicated a more profound loss of genetic diversity as well as a higher risk of inbreeding in the Greek island populations than the Greek mainland breeds. Experimental studies [59] have demonstrated that a higher level of nucleotide diversity is associated with a stronger selection response under stressful conditions. Furthermore, Vilas et al. [60] reported that the high adaptive potential of a population is better indicated by the allelic diversity of neutral markers than by expected heterozygosity. Here, we investigated cattle breeds, whose geographical origin ranged from the cattle domestication center to the most western and the most northern parts of Europe. The overall diversity estimators suggest higher allelic diversity in breeds from the region of South East Europe up to Anatolia. However, this general trend is interrupted by some highly fragmented and inbred Greek cattle breeds (see Additional file 3: Figure S1), especially by island breeds such as CRT, AGT, NSY and KAS which are represented by samples of the last remaining indigenous animals. In complete accordance with the strong effect of genetic drift, the corresponding average frequency of private alleles (*fpA*; Table 1) reached the highest value in the Greek island breeds. Comparable high *fpA*-values were observed only in genetically isolated island breeds such as MNRQ and MALL (Table 1). We observed a general trend that characterizes the fragmented breeds at risk of extinction, i.e. that they are highly inbred with a small number of high-frequency private alleles. This is reflected in the high positive correlation between *F* and *fpA* (*r*_*(F, fpA)*_ = 0.72) and low negative correlation between *F* and *npA* (*r*_*(F, npA)*_ = − 0.21).

As outlined in the Background section, all indigenous breeds of South East Europe, Greece, Cyprus, and Anatolia are either under weak artificial selection or not under any kind of coordinated artificial selection. However, there are substantial differences with respect to isolation. The estimated *D*_*EST*_ levels (see Additional file 5: Table S4) suggest a substantial genetic differentiation of the Greek and Cyprus group compared to the remaining groups. This high differentiation is attributed to the genetic drift and inbreeding in the highly fragmented and naturally isolated island breeds (CRT, AGT, NSY, and KAS) and is confirmed by the low differentiation between animals within these breeds (see Additional file 6: Figure S3).

However, empirical studies [59] provide evidence that, rather than inbreeding, genetic drift led to a reduction in diversity in comparable scenarios. Moreover, purging in stressful environments (i.e. natural selection) can maintain a higher diversity level than expected by inbreeding.

In general, each island of the Aegean represents a different entity with a variety of environmental and socioeconomic factors that affect the livestock populations [61]. However, the cattle populations from the Greek islands have constantly suffered from declining population sizes during recent decades. In the case of the CRT breed, even 60 years ago, the statistical data for livestock numbers of indigenous cattle in Crete identified only 149 of the 1954 (7.6%) recorded animals as indigenous or local, which is a rather small percentage [62]. This statistic also documents a very small number of animals for such a large island (one cow per 4.23 km^2^). For the whole territory of the Dodecanese islands, on which the AGT, NSY, and KAS breeds are raised, the same source identifed 2434 of the 6210 recorded animals (39%) as local. Today, the population size of all the above-mentioned cattle breeds is very small (see Additional file 1: Table S3 and Additional file 2). In addition to small population sizes, genetic drift is strengthened by geographical isolation that further hampers animal exchange. Thus, in such cases, inbreeding and genetic drift are unavoidable factors that shape the diversity of these populations. More generally, there is an opposite trend between the economic significance of cattle and of small ruminants when the regions along the line from the Aegean islands toward the Greek mainland, Balkan and Middle Europe are compared.

In contrast to the island breeds, the lowest levels of genetic differentiation (*D*_*EST*_) were observed for the breed groups from Minor Asia and South East Europe and for some mainland Greek breeds [e.g. GRB, PRG, SYK, and KTR (see Additional file 5: Table S4)]. This low differentiation is accompanied by a high level of allelic diversity including a large number of low-frequency private and semiprivate alleles. Low differentiation and high diversity are probably the consequences of a low artificial selection pressure combined with low genetic drift in effectively large (Table 1) and less isolated populations.

All supervised clustering methods applied in this study clearly reveal the level of genetic differentiation. The highly differentiated breeds show long branches in the neighbor network (Fig. 3), neighbor joining tree (see Additional file 7 Figure S4) and maximum likelihood tree (Fig. 4). This is independent of the evolutionary sources of the differentiation, i.e. the artificially selected and artificially isolated breeds (e.g. beef breeds from the UK) and the naturally isolated and naturally selected breeds (e.g. Greek island breeds) show very long branches. However, breeds that are under strong random sampling (island breeds) show even longer branches especially in the ML analysis. On the contrary, for local breeds with a low differentiation and high diversity level, the three methods reveal short branches or even no branches (Figs. 3 and 4) and see Additional file 7: Figure S4. The unsupervised clustering methods provide a partly different tale. Most of the animals from the artificially unselected breeds of South East Europe, Greece, Cyprus and Anatolia remain unclustered with the admixture approach, even at very high *K*-values (Fig. 6) and see Additional file 8 Figure S5. The CRT breed which has the highest inbreeding level (0.457) among the 114 breeds investigated here was the first unselected breed that was clearly clustered with the admixture approach at *K* = 7. The *K*-value of 78 is associated with the smallest cross-validation error and should represent the true number of clusters in our design. Among the aforementioned unselected breeds, we observed clear clustering for the island breeds and the most inbred Buša breed BHB only, which are characterized by high inbreeding. We observed a similar trend regarding clustering for the Greek KTR, SYK, ROG and PRG populations but in different proportions, which reflects their isolation status (see Additional file 2 and (Fig. 1). The remaining unselected breeds and/or animals remained as an unresolved mixture even at the *K*-value with the lowest cross-validation error.

The MDS projection places almost all the unselected breeds from Minor Asia, Greece and Cyprus, and geographic breed groups from South East Europe and East Podolian, in the overlapping space with suggestive trends (Fig. 5), i.e. Anatolian and Cyprus breeds and the Greek island populations KAS and AGT close to the root of the tree and to GIR. The remaining breeds that were sampled in Greece were placed among the above-mentioned island breeds, the South East European Buša and some Italian local breeds. Thereby the breeds that are geographic neighbor of each other overlap in the MDS presentation. Only the KEA breed, which is the historical product of a cross between indigenous animals similar to the modern-day breeds of Brachyceros and some Alpine breeds (OBV, BBV and TGV), is placed between the Alpine and Tyrrhenian breeds, which is also confirmed by the admixture results (see *K* = 8 to 26 in Figure S5 [see Additional file 8 Figure S5].

Estimating the *LD*-based effective population size (see Additional file 4: Figure S2) for different time intervals during the evolution of cattle provides an interesting insight into their demographic history. For example, the pattern of *Ne* at the time of domestication (~ 2000 generations ago) suggests that compared to western European breeds, modern South East European cattle breeds had larger founder population sizes as a result of their proximity to the center of domestication. In fact, up to ~ 50 generations ago, cattle breeds from southeastern Europe had larger *Ne* than those from northwestern Europe. However, the current *Ne* indicates that the population size of the South East European cattle breeds such as Buša and GRB, has decreased substantially in the last 50 generations or so. Furthermore, this observation implies that such populations have accumulated a small number of common but long haplotypes, which contribute to the high *LD*. The industrial revolution, modern breeding practices and changes in customers’ preference have led to the replacement of local cattle breeds with commercial cattle breeds, which mostly originate from northwestern Europe. This factor, coupled with uncontrolled breeding in the remaining fragmented populations due to the absence of modern reproduction and breeding management practices contributed to population shrinkage of most of the previously highly diverse cattle breeds from southeastern Europe.

Based on Fig. 5 and Additional file 8: Figure S5, and based on the results of the previous studies (e.g. [34, 63]), we assume that introgression of indicine ancestry into Anatolian and some Mediterranean cattle occurred. To test this putative indicine introgression, which could have occurred along the migration route from Anatolia, Greece and South East Europe to the southern foothills of the Alps and North West Europe, we calculated the D-statistics. For all the breeds from these areas (Minor Asia, Greece and Cyprus, South East Europe and East Podolian) except KEA and also all the Italian Podolian breeds and some other breeds from South and central Italy, we obtained significant D-statistic values (Fig. 7). The first breed from the southern Alps for which no significant *Bos indicus* influence was found is SIC (Z < |3|), with a D-value close to zero. The D-values clearly decrease as the spatial distance to the origin of *Bos indicus* increases. Interestingly, three breeds from the East Podolian roup also show a significant indicine introgression, among which UKP that was sampled east of the Carpate Mountains. As discussed recently by Verdugo et al. [35], indicine introgression started ~ 4000 years ago and may have been stimulated by the onset of a period of increased aridity known as the 4.2-thousand-year abrupt climate change event. The increased level of diversity in the breeds from South East Europe, and in the Anatolian and some Greek cattle breeds could be, also, the result of various demographic events, including the introgression of *Bos indicus* alleles. It should be noted that we cannot distinguish the proportion of diversity caused by introgression from that caused by other evolutionary forces, e.g. low pressure of artificial selection. We also note that some estimators of allelic diversity, such as the number of private and semi-private alleles measures the proportion of unique alleles not present even in GIR or other neighbor breeds. The fact that we sampled and genotyped some of the close neighboring breeds intrinsically reduced the number of private and semi-private alleles. However, although GIR and many neighbor breeds from South East Europe and Greece were sampled (Fig. 1), the numbers of private and semi-private alleles remained larger than for breeds from other parts of Europe.

Conservation of genetic diversity for sustainable development must be understood as a global long-term task. The repeatedly confirmed evolutionary trade-off hypothesis [59, 64] suggests that increased fitness in the environment of selection is accompanied by a decrease in fitness in other environments. This trade-off is applied for high-performance breeds that are adapted to benign (temperate) environments and for breeds that are adapted for production in stressful environments. In spite of the above-mentioned trade-off, replacement of well-adapted local breeds by high-performance breeds and/or animals from their crosses is forced by the free-market mechanism that intrinsically causes the extinction of local breeds [65]. This replacement process has either already reached the terminal stage or is close to reaching it in many regions [14]. If we consider animal diversity as a globally shared-resource and as a prerequisite for sustainable development in the changing environments, then gradual depletion of neutral diversity present in local breeds under soft selection pressure is a kind of “*tragedy of the commons*” [65, 66]. Therefore, conservation breeding programs must be seen as a regulated long-term exploitation of common resources.

As already discussed above, most probably, because of an abrupt climate change event, farmers started to cross taurine cattle with *Bos indicus* [4, 9] during the early Bronze Age. Such crosses increased the already high level of diversity of local soft-selected breeds. The subsequent long-term adaptation to local environments shaped the bovine mosaic genomes with an unknown but low proportion of indicine alleles in modern-day cattle breeds from Anatolia up to the southern foothills of the Alps. Therefore, conservation of a high diversity level in these partly fragmented breeds could offer valuable genetic resources for future human needs and future abrupt or gradual climate-change events.

## Conclusions

The phylogenetic patterns derived from genome-wide information were quite consistent with the geographic positions and the historical information regarding crossbreeding between Greek and Cyprus cattle. All investigated Cyprus and Greek breeds present a complex mosaic genome of historical and recent admixture events between neighbor and well-separated breeds. While the contribution of some mainland breeds to the pool of genetic alleles seems important, some island and fragmented mainland cattle strains suffer from a severe decline in population size and a loss of alleles due to strong bottlenecks and genetic drift. However, in spite of a markedly reduced genetic diversity level in most island breeds, these show high fertility and longevity in stressful environments. Conservation programs that are a compromise between what is feasible and what is desirable should focus not only on the still highly diverse mainland breeds but also promote and explore the conservation possibilities for island breeds.

## Supplementary information

**Additional file 1: Table S1.** Sample description, group allocation, RGB (color) code assigned to the pre-defined groups within this paper, breed names, breed code, number of sampled and genotyped individuals (N), number of genotyped and unrelated individuals used to estimate the diversity parameters (Nd), current breeding purposes as well as sporadic or recent past breeding purposes in parenthesis, breed origin, and source of the samples or genotypes used in this study or from previous studies [17, 25, 28, 30, 34, 35, 67–70]. **Table S2.** Phenotypic, productive and reproductive traits. Description of phenotype, productive and reproductive traits of the 11 Greek and Cypriot analyzed breeds. **Table S3.** Evolution of bovine population sizes on the islands during the last 60 years. Statistical information regarding the evolution of cattle population on the Greek island based on [62].

**Additional file 2.** Detailed description of the indigenous Greek and Cypriot cattle. Compilation of information on Greek indigenous cattle breeds from diverse sources [16, 33, 71–87]. This includes also information from records in Greek, which were formerly not accessible to the international scientific community due to language barriers.

**Additional file 3: Figure S1.** Tessellated projection. Spatial geographic presentation of the herein estimated diversity parameters (*mA, AR, H*_*E*_*, H*_*E(SNP)*_*, npA, nspA*).

**Additional file 4: Figure S2.** Tessellated projection. Spatial geographic presentation of the estimated effective population number (*Ne*_*5*_, *Ne*_*50*_, *Ne*_*2000*_).

**Additional file 5: Table S4.** Genetic differentiation index. Pairwise values for *D*_*EST*_, per analyzed breed. Maximum and minimum values within each of the predefined geographical groups are depicted in bold letters in gray rectangle. Maximum and minimum values considering all 115 breeds are in bold letters in green and yellow, respectively.

**Additional file 6: Figure S3.** Tessellated projection. Spatial geographic presentation of the estimated allele sharing distance matrix (D_PS_) among breeds using multi-allelic SNP-blocks.

**Additional file 7: Figure S4.** Phylogenetic tree. Neighbor-joining tree based on Nei’s genetic distance **D**_**A**_ using multi-allelic SNP blocks. Mongolian yak (YAK) was used as a root. Dotted lines indicate the reduced length of YAK and GIR to improve visibility. Special square marks represent the influence of East-Podolian (grey), Alpine (green) and North-West (olive green) group.

**Additional file 8: Figure S5.** Admixture analysis. Population structure of the 114 studied breeds/populations presented for each of *K* inferred ancestral population value, using a genome‐wide set of bi-allelic SNPs. All the analyzed individuals are presented as colored vertical bars, divided at most *K* segments and with proportional heights, according to their genotype membership. In the depicted plots (*K *= 2 to *K *= 26), a different color is presented for each cluster. From left to right within each group the presented breeds are as follows: Minor Asia: ATER, ATBC, ATSR, ATSY, TRG; Greece and Cyprus: CYP, AGT, CRT, NSY, GRB, KAS, KEA, PRG, ROG, SYK, KTR; South East Europe: RHS, MKB, SRB, PRE, RMB, SHB, DGB, DBB, MAB, LKB, SKB, MNB, BHB, HRB; East Podolian: HRI, HRP, UKP; Tyrrhenian: PODO, CINI, MOSI, RSIC, MOSA, SARD, SBRU, CORS, AGER, MARE, CHI, MPIS, CALV, MCH, RMG, GARF, PONT, MODE, CABA, REGG, PMT, BURL; Alpine: PRDO, OVAR, REND, BPUS, PUST, SIC, PIN, TGV, MWF, OBV, BBV, DFV, FGV, VOG, ABO, MON, TAR; France: RDBI, SAL, AUB, LIM, CHR, PAR, BAQ, GAS; Iberian: MNRQ, MALL, NGAN, CANA, MARI, ALEN, BAR, MARO, SYG; North West Europe: BPN, NOR, MAN, BBB, LKF, HF, GNS, JSY, HER, SHR, KRY, DXT, GLW, AAN, HGL, NRC, SERC, FJL, FIAY, FINE, FINW, FINN, YARO.

## Data Availability

The datasets used and/or analysed during the current study are available from the corresponding author on reasonable request.
