# Supplementary material for: Genomic diversity and population structure of the indigenous Greek and Cypriot cattle populations
Source: Genet Sel Evol. 2020 Jul 29;52:43. doi: 10.1186/s12711-020-00560-8 (PMC7391618; doi:10.1186/s12711-020-00560-8)
Supplement: Supplementary file 1 — Additional file 1: Table S1. Sample description, group allocation, RGB (color) code assigned to the pre-defined groups within this paper, breed names, breed code, number of sampled and genotyped individuals (N), number of genotyped and unrelated individuals used to estimate the diversity parameters (Nd), current breeding purposes as well as sporadic or recent past breeding purposes in parenthesis, breed origin, and source of the samples or genotypes used in this study or from previous studies [17, 25, 28, 30, 34, 35, 67–70]. Table S2. Phenotypic, productive and reproductive traits. Description of phenotype, productive and reproductive traits of the 11 Greek and Cypriot analyzed breeds. Table S3. Evolution of bovine population sizes on the islands during the last 60 years. Statistical information regarding the evolution of cattle population on the Greek island based on [62]. [file 12711_2020_560_MOESM1_ESM.docx]

**Additional file 1 Table S1 Sample description**

| **Group** | **RGB code (color)** | **Breed** | **Code** | **N** | **Nd** | **Breeding purposes** | **Breed origin** | **Source of genotypes** |
| --- | --- | --- | --- | --- | --- | --- | --- | --- |
| **Out-Groups** | 43/0/0 (black) | Yak | YAK | 26 | 26 | milk, work, beef | Mongolia | [68] |
|  |  | Gir (Zebu) | GIR | 30 | 24 | milk, work, beef | India (Brasil) | Eggen, pers comm., [35] |
|  |  | N'Dama | NDA | 42 | 27 | milk, work, beef | Burkina Faso/Guinea | Eggen, pers comm., [35], [69] |
| **Minor Asia** | 212/0/170 (pink) | Anatolian East Red cattle | ATER | 20 | 17 | milk, (beef) | Turkey | [35], [25] |
|  |  | Anatolian Black cattle | ATBC | 43 | 37 | milk | Turkey | [17] |
|  |  | Anatolian South Red cattle | ATSR | 21 | 17 | milk, beef | Turkey | [35], [25] |
|  |  | Anatolian South Yellow cattle | ATSY | 8 | 7 | milk | Turkey | [25] |
|  |  | Turkish Grey cattle | TRG | 8 | 8 | milk, beef, work | Turkey | [70] |
| **Greece and Cyprus** | 0/112/192 (blue) | Cyprus cattle | CYP | 14 | 12 | beef, (work) | Cyprus | This study, [34] |
|  |  | Agathonisi cattle | AGT | 6 | 6 | beef, (work) | Greece | This study |
|  |  | Crete cattle | CRT | 11 | 11 | beef, (work) | Greece | This study |
|  |  | Nisyros cattle | NSY | 7 | 7 | beef, (work, milk) | Greece | This study |
|  |  | Greek Brachyceros cattle | GRB | 116 | 41 | beef, (work, milk) | Greece | This study, [34] |
|  |  | Kastelorizo cattle | KAS | 4 | 4 | beef, (work) | Greece | This study |
|  |  | Kea cattle | KEA | 97 | 27 | beef, milk, (work) | Greece | This study |
|  |  | Greek Prespa cattle | PRG | 10 | 9 | beef, (milk, work) | Greece | This study |
|  |  | Greek Rodope cattle | ROG | 12 | 9 | beef, (work, milk) | Greece | This study |
|  |  | Sykia cattle | SYK | 21 | 17 | beef, (work, milk) | Greece | This study, [35] |
|  |  | Katerini cattle | KTR | 20 | 19 | beef, (work, milk) | Greece | This study |
| **South East Europe** | 112/48/160 (dark blue) | Rhodopean Shorthorn | RHS | 24 | 17 | beef, milk | Bulgaria | [17] |
|  |  | Macedonian Buša | MKB | 42 | 22 | beef, milk | North Macedonia | This study, [17] |
|  |  | Serbian Buša | SRB | 58 | 20 | beef, milk | Serbia | [17], [70], [71] |
|  |  | Albanian Prespa cattle | PRE | 39 | 29 | beef, milk | Albania | [17] |
|  |  | Red Metochian Buša | RMB | 26 | 17 | beef, milk | Kosovo | [17] |
|  |  | Sharri Buša | SHB | 21 | 17 | beef, milk | Kosovo | [17] |
|  |  | Dilagjini Buša | DGB | 21 | 21 | beef, milk | Kosovo | [17] |
|  |  | Dibra Buša | DBB | 25 | 25 | beef, milk | Albania | [17] |
|  |  | Middle Albanian Buša | MAB | 43 | 43 | beef, milk | Albania | [17] |
|  |  | Lekbian Buša | LKB | 27 | 27 | beef, milk | Albania | [17] |
|  |  | Skodra Buša | SKB | 14 | 14 | beef, milk | Albania | [17] |
|  |  | Monte-Negro Buša | MNB | 23 | 19 | beef, milk | Montenegro | [17], [71] |
|  |  | Bosnian Buša | BHB | 18 | 18 | beef, milk, (work) | Bosnia & Herzegovina | [17] |
|  |  | Croatian Buša | HRB | 28 | 28 | beef, milk, (work) | Croatia | [17] |
| **East Podolian** | 128/128/128 (grey) | Croatian Istrian cattle | HRI | 30 | 28 | beef, (work) | Croatia | [17] |
|  |  | Croatian Podolian cattle | HRP | 24 | 24 | work, beef | Croatia | [17] |
|  |  | Ukrainian Podolian cattle | UKP | 24 | 21 | milk | Ukraine | [70] |
| **Tyrrhenian** | 255/212/42 (light yellow) | Podolica | PODO | 25 | 25 | beef, milk, work | Italy | [30] |
|  |  | Cinisara | CINI | 30 | 30 | milk | Italy | [30] |
|  |  | Modicana Sicily | MOSI | 29 | 29 | milk, (beef) | Italy (Sicily) | [30] |
|  |  | Rossa Siciliana | RSIC | 24 | 24 | milk | Italy (Sicily) | [30] |
|  |  | Modicana Sardinia | MOSA | 28 | 28 | milk, beef | Italy (Sardinia) | [30], [34] |
|  |  | Sarda | SARD | 30 | 30 | beef | Italy (Sardinia) | [30], [34] |
|  |  | Sardo-Bruna | SBRU | 10 | 10 | beef, milk | Italy (Sardinia) | [30] |
|  |  | Corsican cattle | CORS | 33 | 30 | beef | France (Corsica) | [34] |
|  |  | Agerolese | AGER | 22 | 22 | beef, milk | Italy | [30] |
|  |  | Maremmana | MARE | 51 | 34 | beef, (work) | Italy | [34], [71], [30] |
|  |  | Chianina | CHI | 18 | 12 | beef | Italy | [69], [71] |
|  |  | Mucca Pisana | MPIS | 23 | 15 | work, beef, milk | Italy | [30] |
|  |  | Calvana | CALV | 24 | 24 | beef | Italy | [30] |
|  |  | Marchigiana | MCH | 23 | 21 | beef, (work) | Italy | [69], [30] |
|  |  | Romagnola | RMG | 54 | 18 | beef | Italy | Eggen, pers comm., [69], [30] |
|  |  | Garfagnina | GARF | 23 | 23 | milk, beef | Italy | [30] |
|  |  | Pontremolese | PONT | 24 | 13 | beef | Italy | [30] |
|  |  | Modenese | MODE | 23 | 23 | beef, milk | Italy | [30] |
|  |  | Cabannina | CABA | 22 | 22 | milk | Italy | [30] |
|  |  | Reggiana | REGG | 26 | 26 | milk | Italy | [30] |
|  |  | Piedmontese | PMT | 34 | 16 | beef, (milk) | Italy | Eggen, pers comm, [69], [30] |
|  |  | Burlina | BURL | 24 | 24 | milk, beef | Italy | [30] |
| **Alpine** | 102/153/0 (green) | Pezzata Rossa D'Oropa | PRDO | 23 | 23 | milk | Italy | [30] |
|  |  | Ottonese-Varzese | OVAR | 43 | 31 | milk, beef, work | Italy | [30] |
|  |  | Rendena | REND | 24 | 24 | milk, (beef) | Italy | [30] |
|  |  | Bará-Pustertaler | BPUS | 24 | 24 | milk, beef | Italy | [30] |
|  |  | Pustertaler | PUST | 24 | 24 | milk | Austria/Italy | [30] |
|  |  | Cika | SIC | 26 | 26 | milk, beef | Slovenia | [17] |
|  |  | Pinzgauer cattle | PIN | 29 | 29 | milk, beef | Austria/Italy | [17] |
|  |  | Tiroler Grauvieh | TGV | 50 | 50 | milk, beef | Austria/Italy | [17] |
|  |  | Murnau-Werdenfelser | MWF | 46 | 46 | beaf, milk | Germany | [17] |
|  |  | Original Braunvieh | OBV | 35 | 35 | milk, beef | Germany/Switzerland | [17] |
|  |  | Braunvieh | BBV | 50 | 50 | milk, (beef) | Germany/Switzerland | [17] |
|  |  | Fleckvieh | DFV | 50 | 50 | milk, beef | Germany/Austria | [17] |
|  |  | Gelbvieh | FGV | 50 | 50 | milk, beef | Germany | [17] |
|  |  | Vosges cattle | VOG | 18 | 18 | milk | France | [28] |
|  |  | Abondance | ABO | 22 | 22 | milk | France | [28] |
|  |  | Montbéliarde | MON | 28 | 28 | milk (beef) | France | [28] |
|  |  | Tarentaise | TAR | 37 | 37 | milk (beef) | France | [17] |
| **France** | 250/140/0 (orange) | Raco di Biou | RDBI | 29 | 29 | beef | France | [34] |
|  |  | Salers | SAL | 26 | 26 | beef | France | [28] |
|  |  | Aubrac | AUB | 22 | 22 | beef | France | [28] |
|  |  | Limousin | LIM | 73 | 48 | beef | France | Eggen, pers comm., [69], [17] |
|  |  | Charolais | CHR | 52 | 39 | beef | France | Eggen, pers comm., [69], [28] |
|  |  | Parthenaise | PAR | 17 | 17 | beef | France | [28] |
|  |  | Blonde d'Aquitaine | BAQ | 35 | 33 | beef | France | Eggen, pers comm., [17] |
|  |  | Gascon | GAS | 22 | 22 | milk | France | [28] |
| **Iberian** | 250/140/0 (red) | Menorquina | MNRQ | 30 | 30 | beef, (work) | Spain (Menorca) | [34] |
|  |  | Mallorquina | MALL | 30 | 30 | beef | Spain (Majorca) | [34] |
|  |  | Negra Andaluza | NGAN | 32 | 14 | beef | Spain | [34] |
|  |  | Casta Navarra | CANA | 30 | 30 | beef | Spain | [34] |
|  |  | Marismeña | MARI | 22 | 22 | beef | Spain | [34] |
|  |  | Alentejana | ALEN | 11 | 10 | beef, work | Portugal | [35], [71] |
|  |  | Barrosa | BAR | 14 | 14 | beef, milk, work | Portugal | [17] |
|  |  | Maronesa | MARO | 20 | 19 | beef, (work) | Portugal | [17], [71] |
|  |  | Sayaguesa | SYG | 11 | 11 | milk | Spain | This study, [71] |
| **North West Europe** | 128/98/0 (green olive) | Bretonne Black Pied | BPN | 15 | 15 | milk, (beef) | France | [28] |
|  |  | Normande | NOR | 30 | 30 | beef | France | [28] |
|  |  | Maine-Anjou | MAN | 20 | 20 | beef | France | [28] |
|  |  | Blanc Bleu Belge | BBB | 45 | 45 | beef | Belgium | [17] |
|  |  | Dutch Belted cattle | LKF | 22 | 22 | milk, beef | Netherlands | [17], [71] |
|  |  | Holstein | HF | 50 | 50 | milk | Germany | [17] |
|  |  | Guernsey | GNS | 31 | 16 | milk | Channel Islands | Eggen, pers comm., [69] |
|  |  | Jersey | JSY | 52 | 49 | milk | Channel Islands | [25], [17], [28] |
|  |  | Hereford | HER | 65 | 41 | beef | England | Eggen, pers comm., [69], [17] |
|  |  | Shorthorn | SHR | 14 | 13 | beef | England | [25] |
|  |  | Kerry | KRY | 16 | 14 | milk | [35] | [35] |
|  |  | Dexter | DXT | 22 | 16 | beef, milk | Ireland | This study, [69] |
|  |  | Galloway | GLW | 40 | 40 | beef | Scotland | [25], [17] |
|  |  | Angus | AAN | 66 | 48 | beef | Scotland | Eggen, pers comm., [69] |
|  |  | Highland | HGL | 28 | 27 | beef | Scotland | [35], [86], [17], [71] |
|  |  | Norwegian Red cattle | NRC | 56 | 34 | milk | Norway | Eggen, pers comm., [69], [17] |
|  |  | Swedish Red cattle | SERC | 25 | 24 | milk | Sweden | [71] |
|  |  | Fjaell cattle | FJL | 24 | 22 | milk | Sweden | This study, [71] |
|  |  | Finnish Ayrshire | FIAY | 53 | 42 | milk, (beef) | Finland | [69], [25], [70] |
|  |  | Eastern Finncattle | FINE | 40 | 20 | milk, beef | Finland | [70] |
|  |  | Western Finncattle | FINW | 40 | 35 | milk, beef | Finland | [70] |
|  |  | Northern Finncattle | FINN | 25 | 18 | milk, beef | Finland | [70] |
|  |  | Yaroslavskaya | YARO | 20 | 20 | milk | Russian Federation | [70] |

Group allocation, RGB (color) code assigned to the pre-defined groups in the paper, breed names, breed code, number of sampled and genotyped individuals (N), number of genotyped and unrelated individuals used for estimation of diversity parameters (Nd), current breeding purposes as well as sporadic or recent past breeding purposes in parenthesis, breed origin and source of the samples or genotypes used in this study (Source).

**Additional file 1 Table S2** **Phenotypic, productive and reproductive traits**

| **Traits** | | **Cattle populations/Breeds** | | | | | | | | | | |
| --- | --- | --- | --- | --- | --- | --- | --- | --- | --- | --- | --- | --- |
|  | | **CYP** | **AGT** | **CRT** | **NSY** | **GRB** | **KAS** | **KEA** | **KTR** | **PRG** | **ROG** | **SYK** |
| **Phenotypic traits** | Withers height (cm) |  |  |  | 120 - 140 | 90 - 110 |  | 126.4 |  |  |  |  |
|  | Male |  |  |  |  |  | 112.5 |  | 123.5 |  |  | 108-120 |
|  | Female |  |  |  |  |  | 101.6 |  | 113.5 |  |  | 106 - 116 |
|  | Stature (cm) |  |  |  |  |  |  |  |  |  |  |  |
|  | Body length (cm) |  |  |  |  |  | 145 - 165 | 142.3 |  |  |  |  |
|  | Body weight, 12 months weight (kg) |  |  |  |  |  |  |  |  |  |  |  |
|  | Male | 342 |  |  | 600 | 300 |  | 500 | 375 (308-434) |  |  | 375 |
|  | Female | 307 |  |  | 450 | 200 - 220 |  | 300 | 280 |  |  | 280 |
|  | Birth weight (kg) |  |  |  |  |  |  |  |  |  |  |  |
|  | Male | 31 |  |  |  | 14 - 16 | 13 |  |  |  |  |  |
|  | Female | 30 |  |  |  |  | 11 |  |  |  |  |  |
|  | Chest girth (cm) |  |  |  |  |  |  | 166 |  |  |  | 151 |
|  | Chest depth (cm: |  |  |  |  | 54 |  | 63.6 | 60.4 |  |  | 60.2 |
|  | Rump length (cm) |  |  |  |  |  |  | 46.7 |  |  |  | 39.9 |
| **Productive traits** | Lactation (days) | Usually not milked |  |  | Usually not milked | 180 | Usually not milked | 150 - 270 | 180 |  |  | 180 |
|  | Milk yield (kg/year) |  |  |  |  | 500 – 1200 |  | 1500 | 500-700 |  |  | 500 |
|  | Fat content (%) |  |  |  |  | 4.5 |  | 3.7 |  |  |  |  |
|  | Protein content (%) |  |  |  |  |  |  |  |  |  |  |  |
|  | Carcass weight (kg) |  |  |  |  | 160 -180 |  | 130-180 | 130-180 |  |  | 130 - 180 |
|  | Dressing percentage (%) |  |  |  |  | 45% |  |  |  |  |  |  |
|  | Other characteristics | Suitable for work, resistance, longevity, adaptability |  |  | Resistance, longevity, adaptability, easy calving | Resistance, longevity, adaptability, easy calving | Resistance, longevity, adaptability, easy calving | Suitable for work, resistance, longevity, adaptability, easy calving | Suitable for work, resistance, longevity, adaptability, easy calving |  |  | Suitable for work, resistance, longevity, adaptability |
| **Reproductive traits** | Sexual maturity (months) | 15 |  |  |  | 12 |  | 12-15 |  |  |  | 12-15 |
|  | Age of mating (months) |  |  |  |  | 18 |  | 17-20 |  |  |  | 17-20 |
|  | Fertility (calves/year) |  |  |  | 1 | 1 |  | 1 | 1 |  |  | 1 |
|  | Breeding time (years) |  |  |  |  |  |  |  |  |  |  |  |
|  | Lifetime (years) |  |  |  |  | 20-25 |  | 15 - 20 | 15-20 |  |  | 15-20 |
|  | Economic maturity |  |  |  |  |  |  |  |  |  |  |  |
|  | Conservation | Endangered-maintained |  |  | Endangered | Endangered-maintained | Endangered | Endangered-maintained | Endangered-maintained |  |  | Endangered-maintained |
|  | Estimated number of individuals | 1384 | 25 | 25 | 25 | 6000-7000 | 10 | 80 | 400 | 150 | 250 | 180 |

**Additional file 1 Table S3 Evolution of bovine population sizes on the islands during the last sixty years (source [63])**

| **Islands** |  | **Number of farms** | | | | **Number of animals** | | | |
| --- | --- | --- | --- | --- | --- | --- | --- | --- | --- |
|  | **Year** | **1951** | **1961** | **1991** | **2001** | **1951** | **1961** | **1991** | **2001** |
| **Agathonisi** |  | - | - | 4 | 3 | - | - | 14 | 30 |
| **Crete** |  | 26,280 | 20,061 | 402 | 226 | 40,374 | 35,058 | 2670 | 2207 |
| **Kastelorizo (Megisti)** |  | - | - | 6 | 3 | - | - | 46 | 26 |
| **Kea** |  | 865 | 987 | 91 | 132 | 1745 | 2544 | 669 | 1504 |
| **Kythnos** |  | - | - | 125 | 54 | - | - | 688 | 309 |
| **Nisyros** |  | - | - | 16 | 26 | - | - | 251 | 590 |
| **Paros** |  | 1012 | 742 | 106 | 221 | 2279 | 1857 | 415 | 1533 |
