# Supplementary material for: Genomic diversity and population structure of the indigenous Greek and Cypriot cattle populations
Source: Genet Sel Evol. 2020 Jul 29;52:43. doi: 10.1186/s12711-020-00560-8 (PMC7391618; doi:10.1186/s12711-020-00560-8)
Supplement: Supplementary file 2 — Additional file 2. Detailed description of the indigenous Greek and Cypriot cattle. Compilation of information on Greek indigenous cattle breeds from diverse sources [16, 33, 71–87]. This includes also information from records in Greek, which were formerly not accessible to the international scientific community due to language barriers. [file 12711_2020_560_MOESM2_ESM.pdf]

Additional file 2 Detailed description of the indigenous Greek and Cypriot cattle

Cyprus cattle (CYP)

Origin, history and sampling: The indigenous Cyprus cattle historically consisted of two distinct native types, namely the “Mesaoria” i.e. of the plain area and the “Paphos” i.e. of the mountain areas. The “Mesaoria” type was characterized by a heavy animal with a thin coat, reddish brown brown-red skin color and whitish color on the abdomen. This type was suitable for the plain areas [72]. The Paphos type was a small animal with a rough coat with colors in all shades of brown, but black animals were also found. This type was adapted to the mountains and hills. Nowadays the above types are considered as a unique population. For the present study, five samples were collected.

Breed standards, productivity characteristics and reproduction: The indigenous Cyprus cattle are characterized by low growth rate and low productivity, while their milk production is only sufficient to feed the calf of each cow [73]. It is assumed that some local cattle were crossed in 1912 with Devon cattle, but the products of these crosses never came into favor among farmers for raising draught cattle [73]. Thus, the use of native cattle remained stable during time, and consisted in their exploitation as a source of power to perform agricultural work (plowing, threshing) but also to transport people and products. Because of the agricultural mechanization after the Second World War, the preservation of these animals became unprofitable. Common characteristics for both types are a hump, a relatively large dewlap, a black tuft at the end of the tail and the white-gray ring surrounding the muzzle. At the bottom of the limbs and towards the base of the claws, the coat is a fading light white/yellowish color.

The animals graze on natural pastures from March to October and are fed with green barley, maize and alfalfa, if they are available. During winter, draught animals are housed in rather primitive stables and a ration of vetches, oats and chopped straw is provided. Breeding is carried out throughout the year, but efforts are made to arrange for cows to calf during the season from January to April when green forage is plentiful. The calves suckle for 6 to 7 months [72].

Breeding status: According to the official records [74], the number of animals in 2016 was 1384, from a former number of 27,500 in 1960 and 52,916 in 1907. These animals are distributed across 88 farms or kept by smallholders.

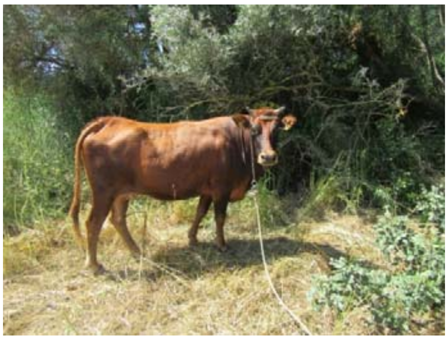
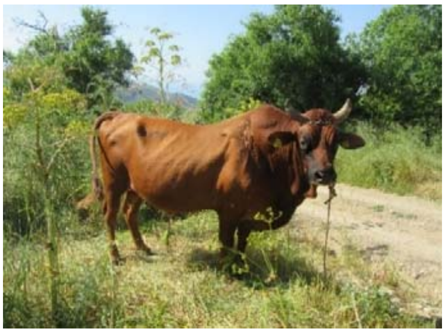

Cyprus cattle (photos provided by I. Bizelis)

Agathonisi Cattle (AGT)

Origin, history and sampling: The animals reared in Agathonisi (an island of the Dodecanese group) are believed to belong to the Greek Brachyceros type with an amalgamation of podolic (steppe) or even Anatolian breed influences [75]. They are well adapted to the island's dry and warm conditions and poor pastures. Crossbreeding with other cattle breeds has not been reported for at least the last 30 years. For the present study, six samples from one herd were collected on the Agathonisi island.

Breed standards, productivity characteristics and reproduction: Height at the withers is about 110–115 cm and body weight ranges from 100 to 150 kg, with bulls reaching 200 kg or more if fed properly. Horns are short to very short, but a few individuals have longer horns. White muzzle rings of variable intensity are present in most but not all animals. Dewlaps occur in several animals. The coat color can take various tones of red to beige with darker, sometimes almost black shadings, but it can also dark brown or black. A distinct lighter or darker ticked gray is also observed. The animals are not milked and they are used only for meat production. Because of the isolation of the Agathonisi island, it is difficult for farmers to buy animal feed and sell livestock products beyond its shores.

Breeding status: A small population was detected in August 2014, which since then has been fluctuating between 25 and 32 individuals spread among four farms in Agathonisi. On the neighboring island, Lipsi, there are also about 20 animals that originated from the same cattle of Agathonisi population.

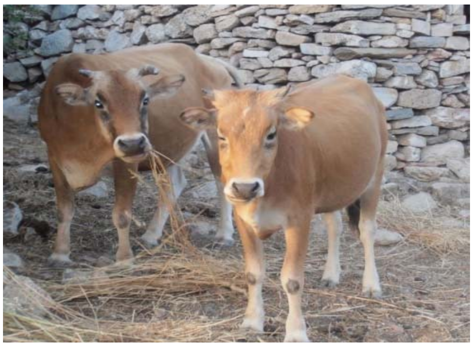
Agathonisi cattle (photo provided by St. Dellepiane)

Crete cattle (CRT)

Origin, history and sampling: These cattle represent the only currently surviving indigenous breed on the island of Crete, derived probably from an ancestral population of cattle bred in Crete for centuries. Old sources [76] report two types of cattle in Crete: (a) the type of Messara (Messara is a plain in the Herakleion prefecture), which was improved through small-scale selection by the breeders, and (b) the mountainous shorthorn type, which is similar to the examined current population. Messara cattle are considered to be extinct today (a detailed report on Messara cattle can be found in [77]). For this study, eleven animals of the mountainous type were sampled from three herds in the Chania region.

Breed standards, productivity characteristics and reproduction: Reared in a dry environment with poor pastures, Crete cattle are considered hardy animals. The breeding purpose was mainly for work. Nowadays, they are bred only for meat. In terms of morphological characteristics, they seem to belong to the shorthorn type. There are currently no complete body measurements of the animals, but the withers mean height is estimated at 115 cm. The mean carcass weight is 118–120 kg.

Breeding status: Today, there are at least 25 animals (6 males and 19 females), which are distributed among four small-scale breeders (AMALTHIA, Network for the Protection of Greek Indigenous Farm Animals, 2018, Bizelis I, personal communication). The largest herd has 15 animals. All cattle descended from a single private herd formed in 1983 with animals bought from the Rodopos peninsula in West Crete.

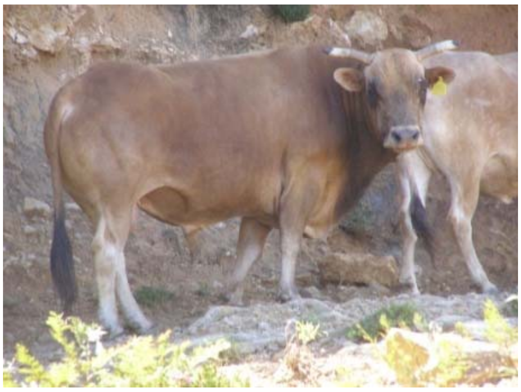

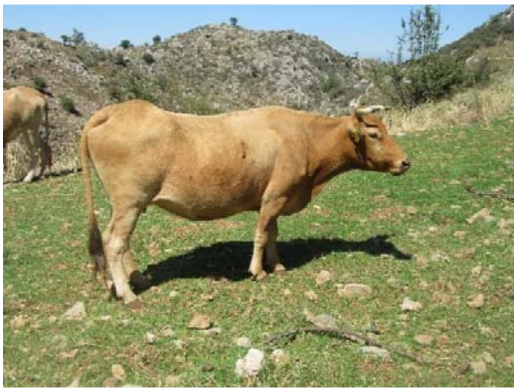

Cow (left) and bull (right) of Crete cattle (photos by V. Lekkas (left) and I. Bizelis (right))

Nisyros cattle (NSY)

Origin, history and sampling: The animals were described as primitive. Their origin is essentially unknown. An often-quoted view is that they originated in Western Asia Minor and arrived with refugees in 1922. According to Manetti [78], cattle from the western coast of Asia Minor that belonged to an unimproved steppe type of low economic value and low productivity were imported into the Archipelagos and the islands. According to the same reference, besides the lighter steppe type, cattle of the "Iberian type", which possibly refers to the shorthorn types of Asia Minor, were also bred in Western Asia Minor. We sampled seven animals for this study.

Breed standards, productivity characteristics and reproduction: In terms of size, they are considerably larger than Agathonisi cattle and also appear to have stronger bodies and a squarer body outline. The height at the withers of adult cows is estimated at 120–130 cm or even reaches 140 cm. Cows usually reach 450–500 kg live weight. Slaughter weight is at ~200–250 kg. Presently, they are not milked. The well-fed bulls can reach a live weight of 600 kg at 2 years. Horns are variable in length and shape. Overall, they are longer and larger/thicker compared to typical short horns but shorter than classic steppe type horns. Body color occurs in ranges from gray/black and solid black, to cinnamon, solid beige/orange, or red/orange with darker facial markings and/or stripes. Some individuals have visible (light) dorsal or eel stripes along the top line/back. Many animals have white rings around the muzzle (mealy markings/deer muzzle).

Breeding status: According to Amalthia records, the population consists of at least 25 animals of both sexes.

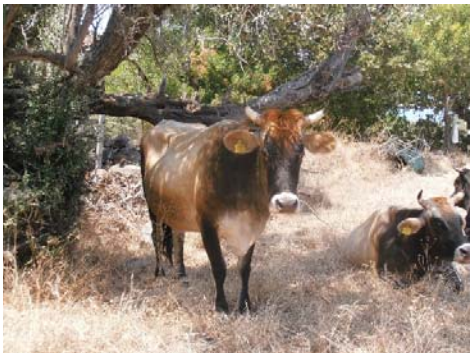
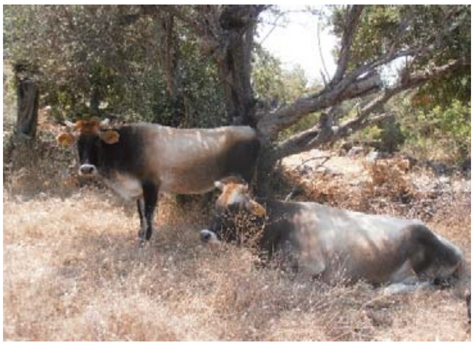

Bulls and cows from a herd of Nisyros cattle (photos provided by St. Dellepiane)

Greek Brachyceros breed (GRB)

Origin, history and sampling: The Greek Brachyceros breed belongs to the group of Illyrian Brachyceros cattle (*Bos taurus brachyceros)*, the most common cattle in the Balkan region in the past [75, 79]. It resembles a dairy type animal [80]. The hardiness of the breed allowed its wide spread in the bovine population of the country. Ninety-seven samples were collected from four farms: (i) Aetochori (Karditsa), (ii) Lepenou (Aetoloakarnania), (iii) Kefalonia island, (iv) Pagoneri (Drama).

Breed standards, productivity characteristics and reproduction: Cows are outdoors on the mountainous poor pastures throughout the year and during the winter, they are usually protected in low-cost stables only in adverse weather conditions. The body size is small with a small head, long neck and small dewlap. The horns are thin with a small forward curve. The trunk is rather short, the chest and thorax have small width. The back and the loin are narrow, too. The pelvis is narrowly inclined. The udders are small and hairy. The overall development of muscles is rather limited. The coat colour is variable (blond, silver-footed, dark-gray, brown, dark brown, black). The colour of the muzzle, horns and hoofs is usually black and there is a white ring around the muzzle (mealy marking). The animals are well adapted and thrifty with a slow growth rate, long life expectancy and high reproductive performance. In the past, the breed was a triple-purpose breed (milk, meat, work). Nowadays, they are reared exclusively for beef production, although the meat production capacity is small. The slaughter age is at 20 months at a slaughter weight of 160–180 kg with a carcass yield of about 45%. The calf is weaned at approximately 6 months.

Breeding status: Nowadays, the breeding areas of Brachyceros cattle are the mountainous areas of Epirus, Thessaly, Aetoloakarnania, Kefalonia Island and Greek Macedonia. The mature reproductive females estimated nearly 7000 animals. In 2016, an Association of Brachyceros cattle breeders was created named the “Greek Shorthorn Cattle Breeders’ Association” [81-83].

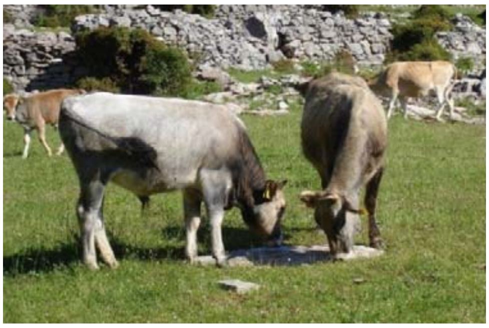
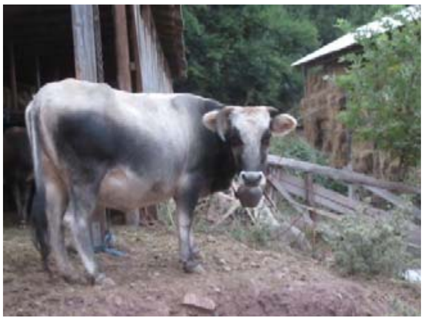
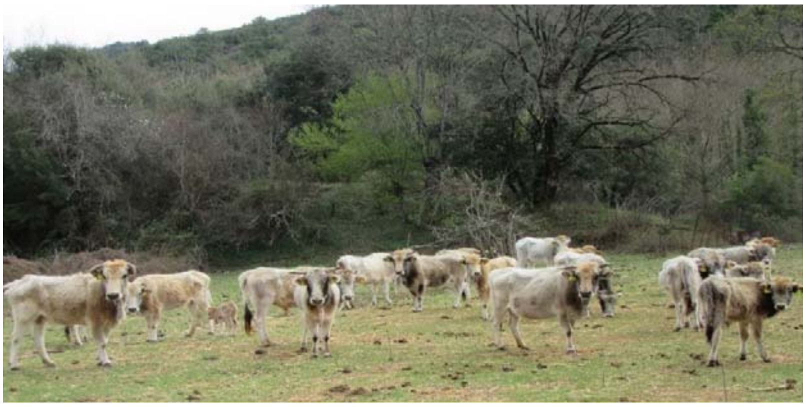

Bulls and cows from a herd of Greek Brachyceros (photos by A. Tsaprailis (above) and I. Bizelis (below))

Kastelorizo cattle (KAS)

Origin, history and sampling: The native cattle of Kastelorizo (Megisti island) constitute a very small population (n = 16 individuals) spread across three different island localities. Temporarily, 3 to 4 animals were kept on the islet of Ro. The animals have been isolated for more than 80 years. There are multiple speculations about their origin. It is possible that the animals' ancestors were imported from the Turkish coast (Western Asia Minor). Another possibility is that they arrived from Astypalea or Kalymnos as many residents of these islands moved to Kastelorizo in the past, along with their livestock and entire households. It is likely that the current animals on the island descended from all above cattle populations. There is no mention of Kastelorizo cattle in Greek bibliographical sources as is the case with most Aegean island cattle. Four samples were collected from two regions of Kastelorizo island (Aheres and Avlonia).

Breed standards, productivity characteristics and reproduction: This population resembles many of the old shorthorn native cattle that were used for work, milk and meat production [84]. The coat color is brown, black or grayish-white with black or white rings around the eyes. The muzzle is black with a white ring. The horns are short, white with black tips turning to the front having a length of approximately 14 cm. The hooves are black and the tail end is brownish to solid brown. The body score of all animals is poor: only three animals were assigned a score of 2 on the scale (1–9), while all other animals were assigned the lowest score of 1 on the scale. The animals are not milked but kept for their meat.

Breeding status: The population is under risk of extinction with only 4 bulls and 12 cows.

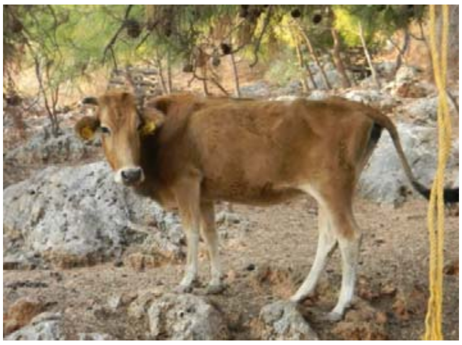
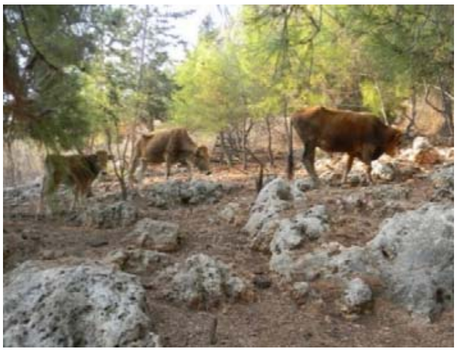

Cow (left) and a bull with cows (right) of Kastelorizo cattle (photos by Y. Achladiotis)

Kea breed (KEA)

Origin, history and sampling: The name of the KEA breed derives from the island of Kea (Cyclades group). Old sources classified the breed as a shorthorn type [75, 77]. The creation of breed, started on 1909, when crossbreeding carried out between local cows and Schwyz bulls [77]. Until the 1930's, bulls from foreign breeds such as Simmental or Sziget were also imported to a lesser degree. The result was the formation of a breed with improved milk production, while maintaining the original characteristics of the Brachyceros breed. Their adaptability as draught animals to the harsh, rocky conditions of the island remained intact [77]. The Kea cattle were larger than the corresponding Brachyceros in mainland Greece (Greek Brachyceros) and the Cretan type (Messaras cattle). After the 1940s, Kea cattle spread to other islands of the Aegean (e.g. Kythnos, Sifnos, Paros, etc.). In Makronissos, the Kea cattle were also well adapted to even tougher environmental conditions with poor grazing and lack of fresh water. In the 1960s, 70 animals were transported to Trifylia in the Peloponnese. During that decade, the modernization of farming in parallel with the intensive efforts to improve the milking potential of the breed led to a gradual reduction in its use. The breed no longer exploited the natural pastures and became more expensive to maintain. In the early 1970s, the above facts led to the inevitable diminishment of the pure breed and its original type.

Ninety-seven samples from six different locations (a. Kea island, b. Makronissos island, c. Kythnos island, d. Paros island, e. Salamina island and f. Trifylia-Peloponnese area) were collected for this study.

Breed standards, productivity traits and reproduction: The coat color fluctuates from blond to dark brown with gray-brown color prevailing. The color is solid with various gradations from light shades mixed with blond to dark or almost black. There are darker and lighter gradations depending on the part of the body and the viewed angle. A white ring encircles the muzzle. The face and the exterior of the ears are darker. Almost all animals have a light dorsal stripe along the spine. The tips of the thin and short horns and the hooves are dark-colored. The coat is thin to moderate and glossy in the summer but coarser in the less-improved animals. The animals are kept for meat and milk and spontaneous for work whereas the animals are well adapted to the island conditions of poor pastures and rocky terrain. The annual milk production is nearly 1500 litters. According to old reports, Kea cows produced on average 16 kg of milk per day immediately after calf weaning [75].

Breeding status: Since 2012, with efforts of Amalthia, the few representative animals discovered on the islands of Kea and Makronissos (4 bulls and 8 cows) have joined a rescue program. The effort has focused on the creation of small nuclei of the breed. The total number of Kea cattle is estimated around 60 to70 animals.

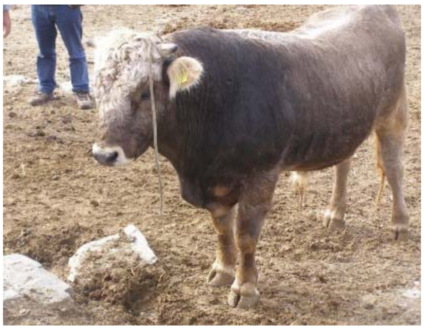

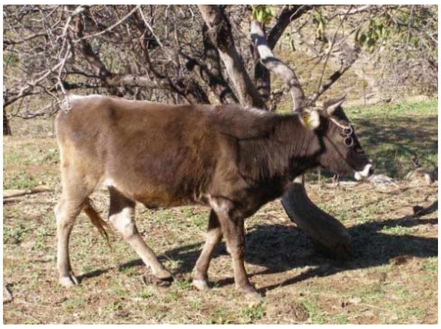

Bull (left) and cow (right) of the Kea cattle (photos by I. Bizelis)

Katerini cattle (KTR)

Origin, history and sampling: Katerini is one of the two Greek cattle strains belonging to the steppe type. The other one is the Sykia cattle. Some similarity is observed between vKaterini cattle and drawings of cattle found in frescoes, on coins and other archaeological findings of the Minoan and Mycenaean periods [81]. The white circle around the muzzle as well as the lyre-shaped horns are characteristic signs of a primitive breed. The animals were selected for draught purposes. The main population was reared in central Macedonia before the 1940s, while another small sub-population was restricted to South Thessaly (Goura type) [75]. The latter population is considered as a more isolated population due to geographical boundaries.

Samples were collected from twenty individuals from two farms in two regions. More specifically: (a) 10 animals were sampled from the Anavra/Goura region (South Thessaly) and(b) 10 animals from the Trikala region (West Thessaly).

Breed standards, productivity characteristics and reproduction: In the past, the breed was used for working purposes. Nowadays, it is used for beef and milk production and sporadically as a draught animal. The breed is medium to large sized with a more developed anterior part of the body and strong legs. The coat colour is gray or gray-silver, mostly dark gray to blackish. The bulls are very dark to solid black without a dorsal stripe. The animals carry long horns with a characteristic lyre shape and black tips. The conformation of the back is often defective with a notable dip between the withers and the posterior. The long tail ends in a dense tuft. The breed is late maturing with the first calving at 29 months. Milk production is low, estimated at 500 to 700 L during a 6-month period. Meat quality is moderate due to the hardness of the muscles. The breed is described as resistant to harsh environmental conditions with an average live weight for males and females of 375 kg and 280 kg and a height of 123 cm and 113 cm, respectively.

Breeding status: The geographic distribution of the breed formerly included the region of Katerini and other plains of north Greece. Nowadays, its breeding area is restricted to the Thessaly plains (Trikala-Kalambaka) and the Anavra/Goura plateau in South West Thessaly. Currently, its population is estimated at less than 400 animals among four registered herds.

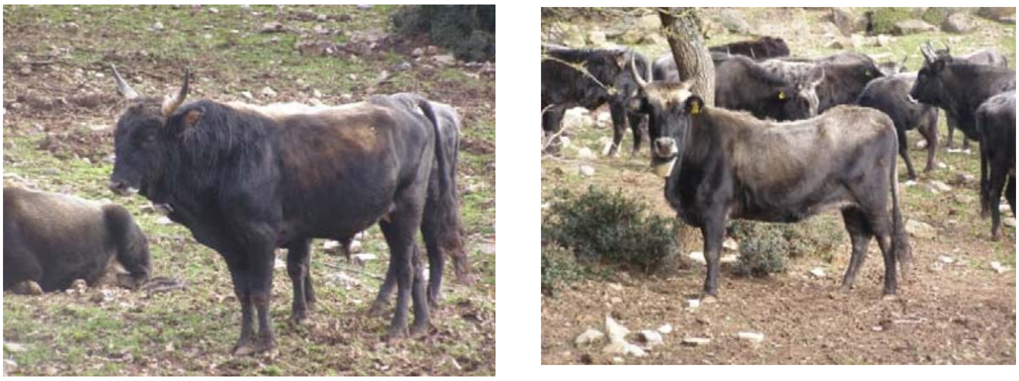

Bull (left) and cows (right) of the Katerini cattle (photos by V. Lekkas)

Greek Prespa Cattle (PRG)

Origin, history and sampling: The Greek Prespa cattle are kept in the homonymous region near the borders with Albania and North Macedonia [82]. Based on their phenotypic characteristics, the animals are attributed to the Greek Shorthorn subtype (Greek Brachyceros).

Ten samples were collected: (a) seven from Agios Achilios, a small island in the Mikri Prespa lake and (b) three from Florina region.

Breed standards, productivity characteristics and reproduction: Colors vary between gray, grayish blue, and reddish or dark brown. The withers height varies between 120–125 cm for males and 95–105 cm for females, while body weight ranges from 230 to 250 kg for males and 120 to 150 kg for females. In the past, they were raised for work, milk and meat but today, only for meat production.

Breeding status: A small population (65 to 70 individuals) is currently found near Prespa (Psarades, Agios Achilios) and in the Florina region [85, 86]. In 2006, 400 to 500 animals of a similar population were recorded in Albania. MtDNA analyses of the aforementioned Albanian population confirmed its classification as one of the Buša breeds, which is of particular conservation importance in the Balkan countries [16].

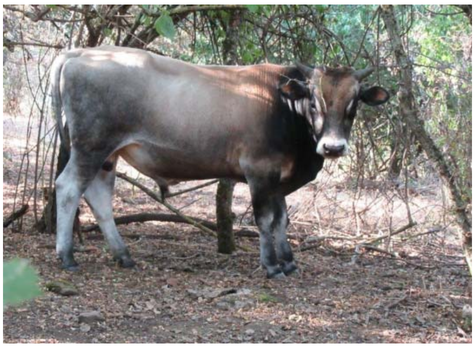

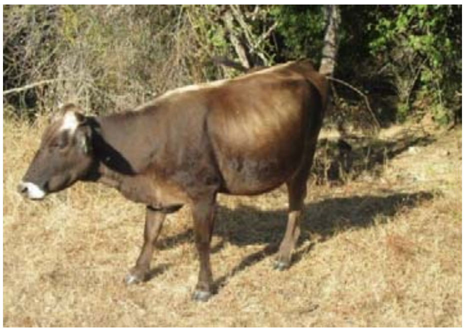

Bull (left) and cow (right) of the Greek Prespa cattle (photos by I. Bizelis)

Greek Rodope Cattle (ROG)

Origin, history and sampling: Greek Rodope cattle are small shorthorn animals. Officially, they are not a recognized breed, although they are reported as a distinct population from the middle of previous century. The Brachyceros, Buša and Anatolian-Podolian types of breeds are considered to have contributed to the creation of the Rodope breed. Psaltis [87] differentiated the population into mountain and plains forms of Greek Thrace. The mountain type was largely kept by the Pomak minority but also in the Greek villages of the Rhodope prefecture, hence its name. It was smaller than the plains' type and darker including black. Nowadays, the population has survived in the area of the Rhodope mountains. During the last 40 years, the breed was crossed with European cosmopolitan breeds and became nearly extinct. The breed was used for work, milk and meat production. Twelve samples were taken from one herd in the Lagada region north of Thessaloniki.

Breed standards, productivity characteristics and reproduction: Greek Rodope cows have coats of red-brown to yellow color, but various shades of dark brown are also found. The bulls are of dark color, commonly black. The withers height of a cow is 100–110 cm with an average body weight ranging from 200 to 250 kg**.** The average body weight of a bull is around 400 kg.

Breeding status: The population is considered as threatened by extinction because there are only 250 animals in a pure herd of the original breed type.

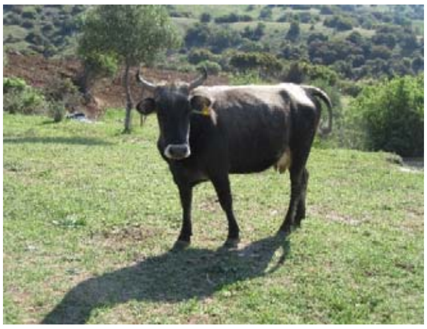
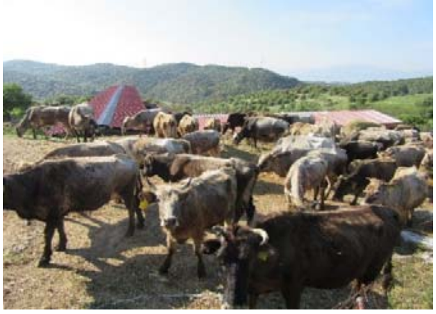

Greek Rodope cattle (photos by I. Bizelis)

Sykia cattle (SYK)

Origin, history and sampling: Sykia is the second of the two Greek strains belonging to the steppe type. However, it is not a typical representative of the Podolian type. According to earlier reports, admixture with the Brachyceros type occurred, resulting in a Greek steppe strain smaller than the other Podolian breeds but larger than the Greek Brachyceros. Its name derives from Sykia village in the Sithonia region of Chalkidiki [88].

Samples from sixteen animals from one breeder (village Sochos, central Macedonia) were collected.

Breed standards, productivity characteristics and reproduction: The breed has a small body size with the withers height in cows and bulls from 106 to 116 cm and from 108 to 120 cm, respectively. The body weight of cows and bulls is 160–240 kg and 180–280 kg, respectively [20]. The primitive appearance is obvious as the front part of the body is more developed than the back. The animals are strong, resistant, thrifty and well-adapted to the extensive farming conditions for exploitation of the poor vegetation in the semi-mountainous areas. The horns are long, lyre-shaped, or crowned with light color at the base, while the tips are black. They are cylindrical and relatively thin, of medium length in bulls and longer in cows. The length of the outer curvature of the horns ranges from 27 to 47 cm.

The coat color varies from silver-gray to dark-black with mixed yellow-brown hair. Darker shades of hair exist on the cheeks, the neck, the front legs, the lower abdomen and the back legs from the pelvis down. There is a lighter coloration on the ribs, the forehead, the nose, the paralumbar fossa and the rump. On the back, a dark stripe is often present. The muzzle and the hooves are black. There is a silver-grayish ring around the nose. The skin is loose in a dewlap with several folds in the bulls. The tuft of the tail is darker in color. The animals were used for work (e.g. plowing, sowing, transporting etc.). Nowadays, they are bred exclusively for meat. Although milk yield is low, additional milking in the spring can give on average 4 kg of milk per day additional to the amount consumed by the calf.

Breeding status: Until 1923, there was a breeding center for Sykia cattle, which provided animals for draught purposes throughout the peninsula of Chalkidiki including Mount Athos. At that time, the number of animals was ~13,000. After 1922, the extension of arable land and the resulting shrinking pastures led to a decrease in the number of cows in the area. According to the breeding center, only 1330 animals were left in 1936 [88]. Foreign improved breeds, which were better suited to intensive breeding, gradually filled the gap caused by the declining numbers of this ancient local breed, and this practice has continued until today.

Until recently, the Sykia cattle strain was considered to have disappeared. In 2005, only a few females remained in four flocks. In 2008, a small nucleus of 80 individuals was discovered in Olympiada, Chalkidiki, by Amalthia. Today, there is also a farm with Sykia animals in the village of Sochos (80 individuals), a farm near Ierissos (40 individuals) and a nucleus consisting of 10 animals in Perdika (Epirus). The breed was genotyped using 19 microsatellites including 20 breeds [33]. The results showed that the subgroup of Mediterranean cattle breeds including Sykia cattle may have had more recent input from the Near East and Africa.

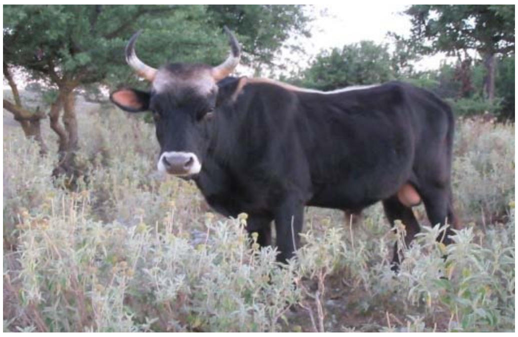 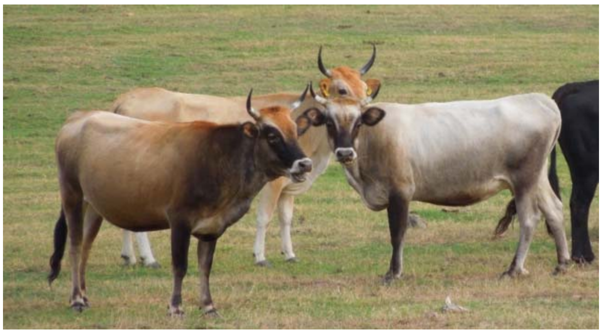

Bull (left) and cows (right) from the Sykia breed (photos by I. Bizelis (left) and P. Koutsouli (right))
