# Supplementary figures and images for: Genomic diversity and population structure of the indigenous Greek and Cypriot cattle populations
Source: Genet Sel Evol. 2020 Jul 29;52:43. doi: 10.1186/s12711-020-00560-8 (PMC7391618; doi:10.1186/s12711-020-00560-8)

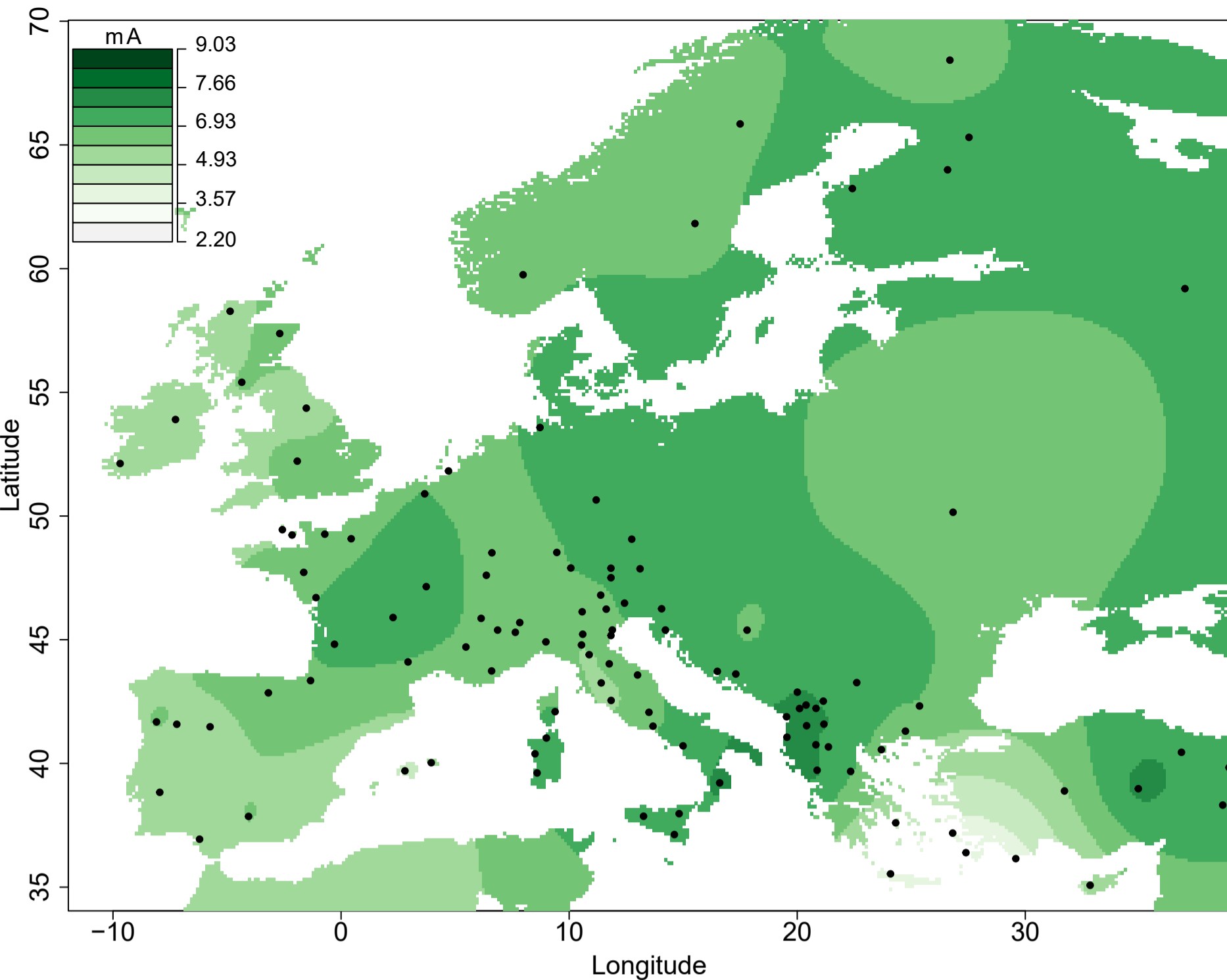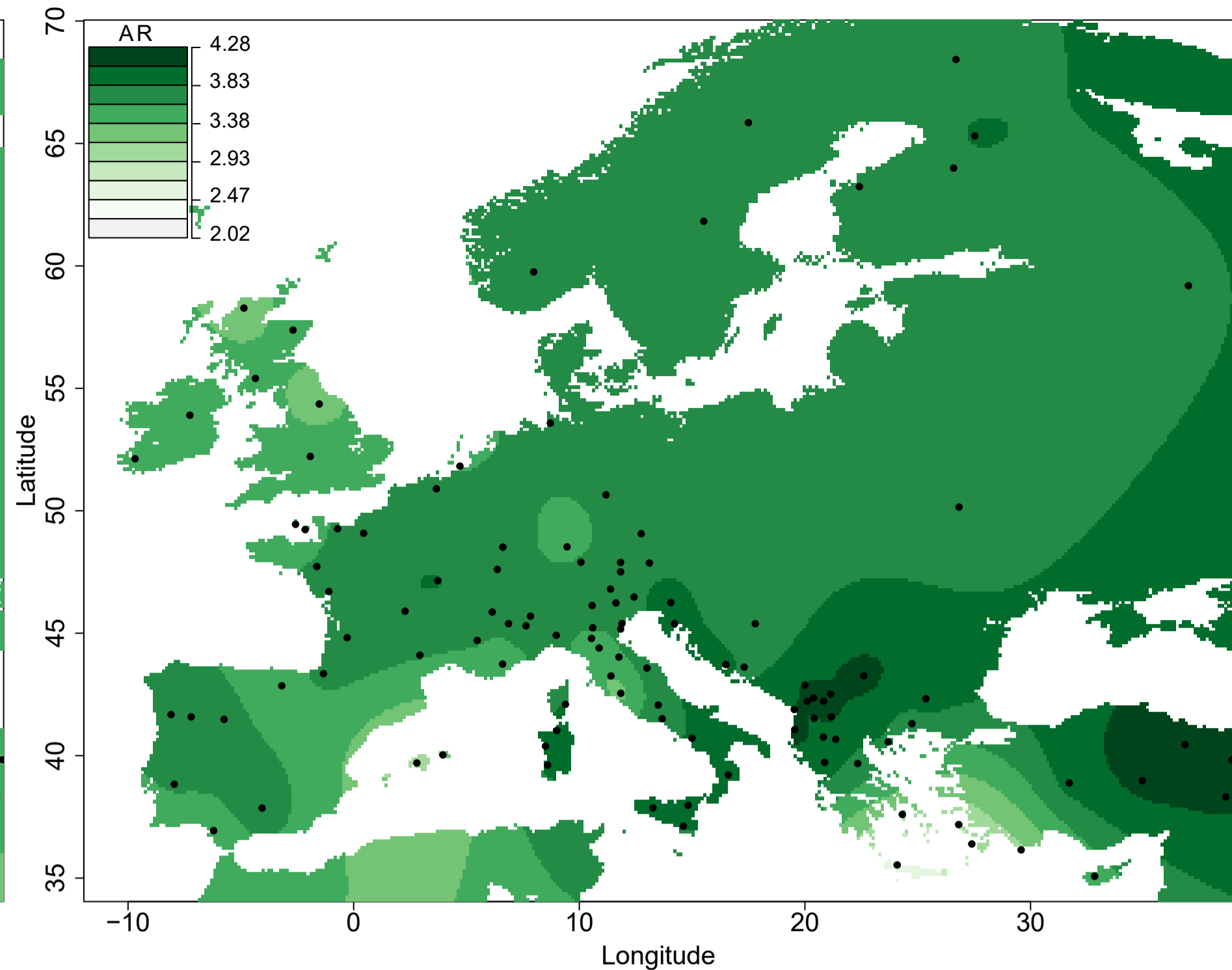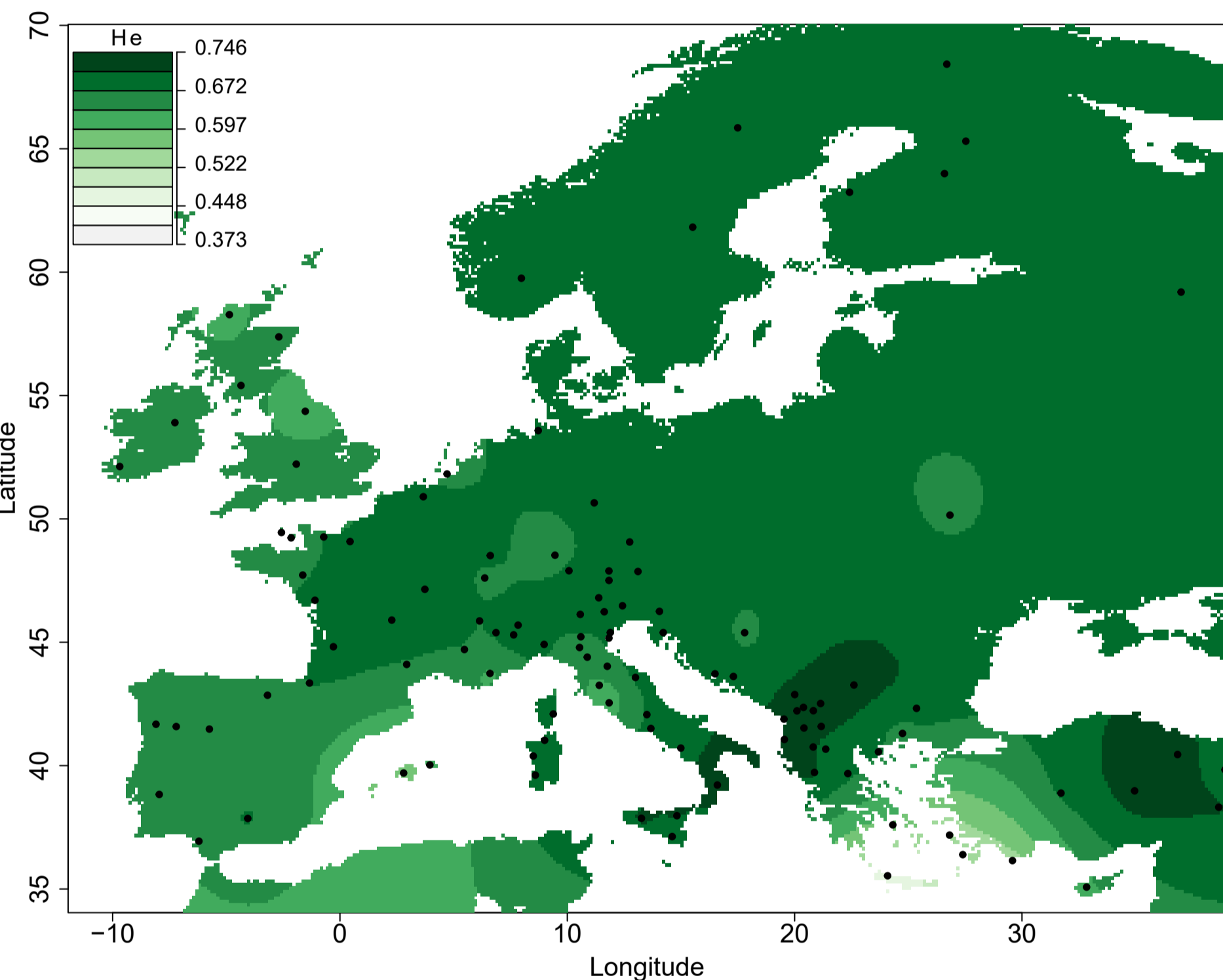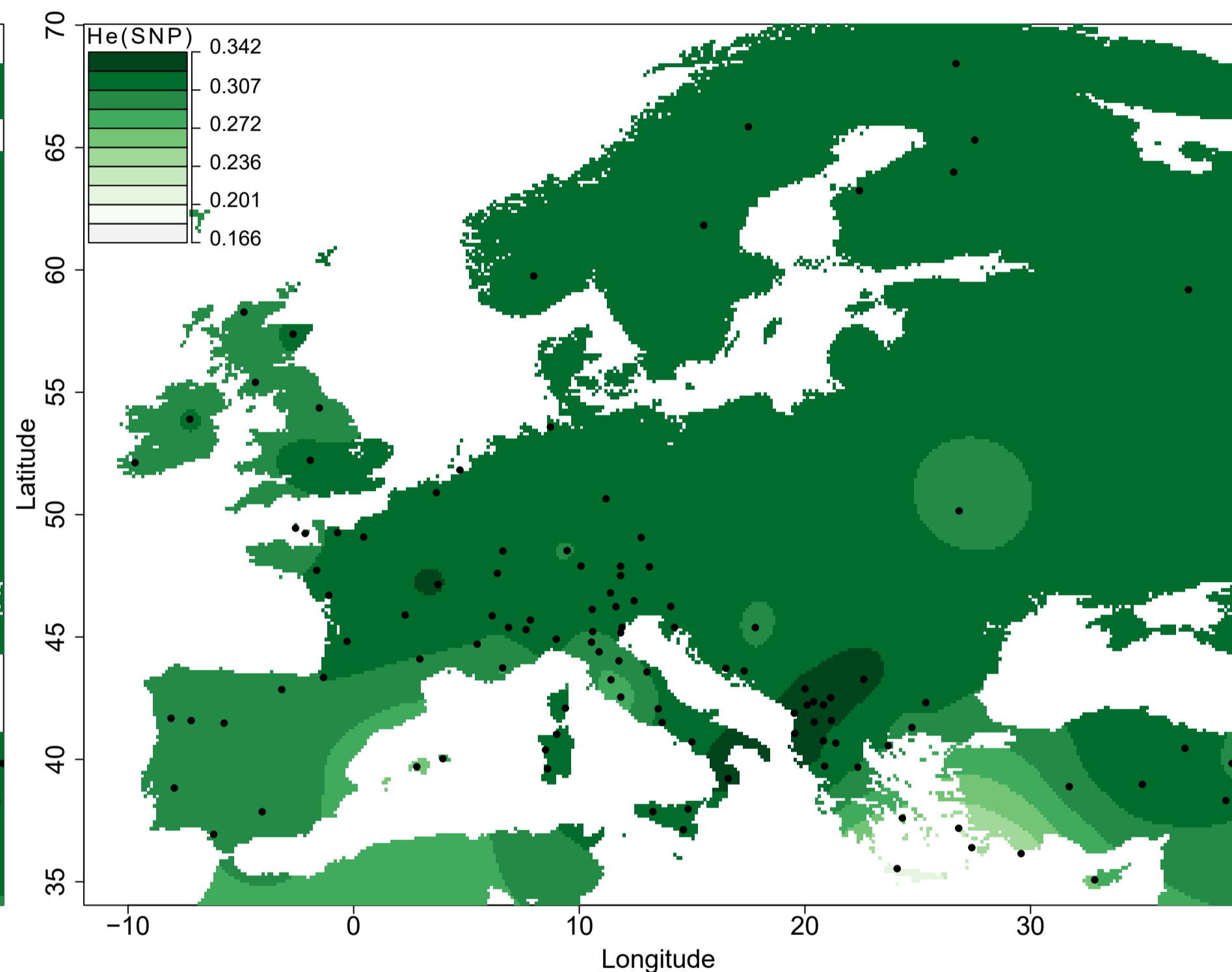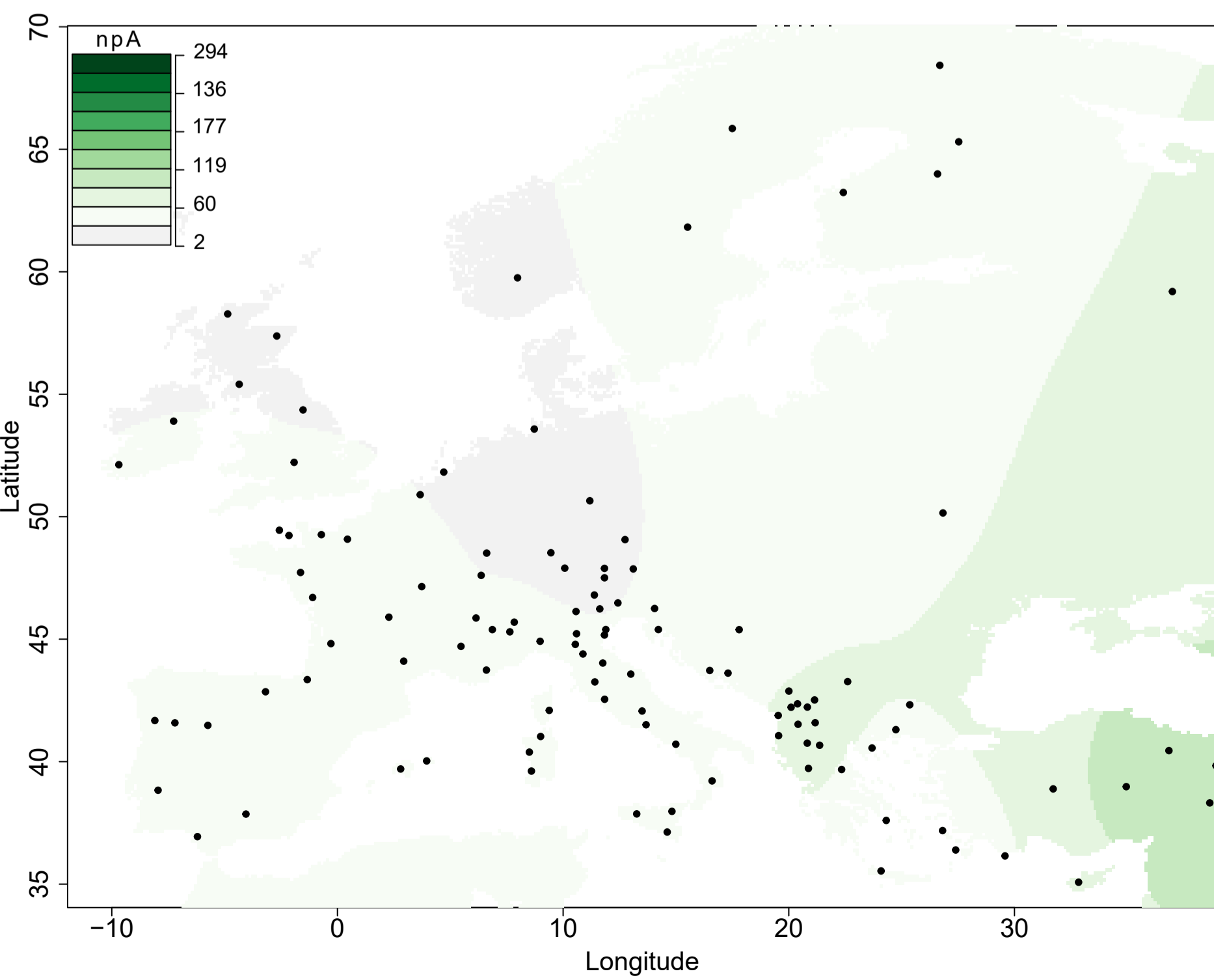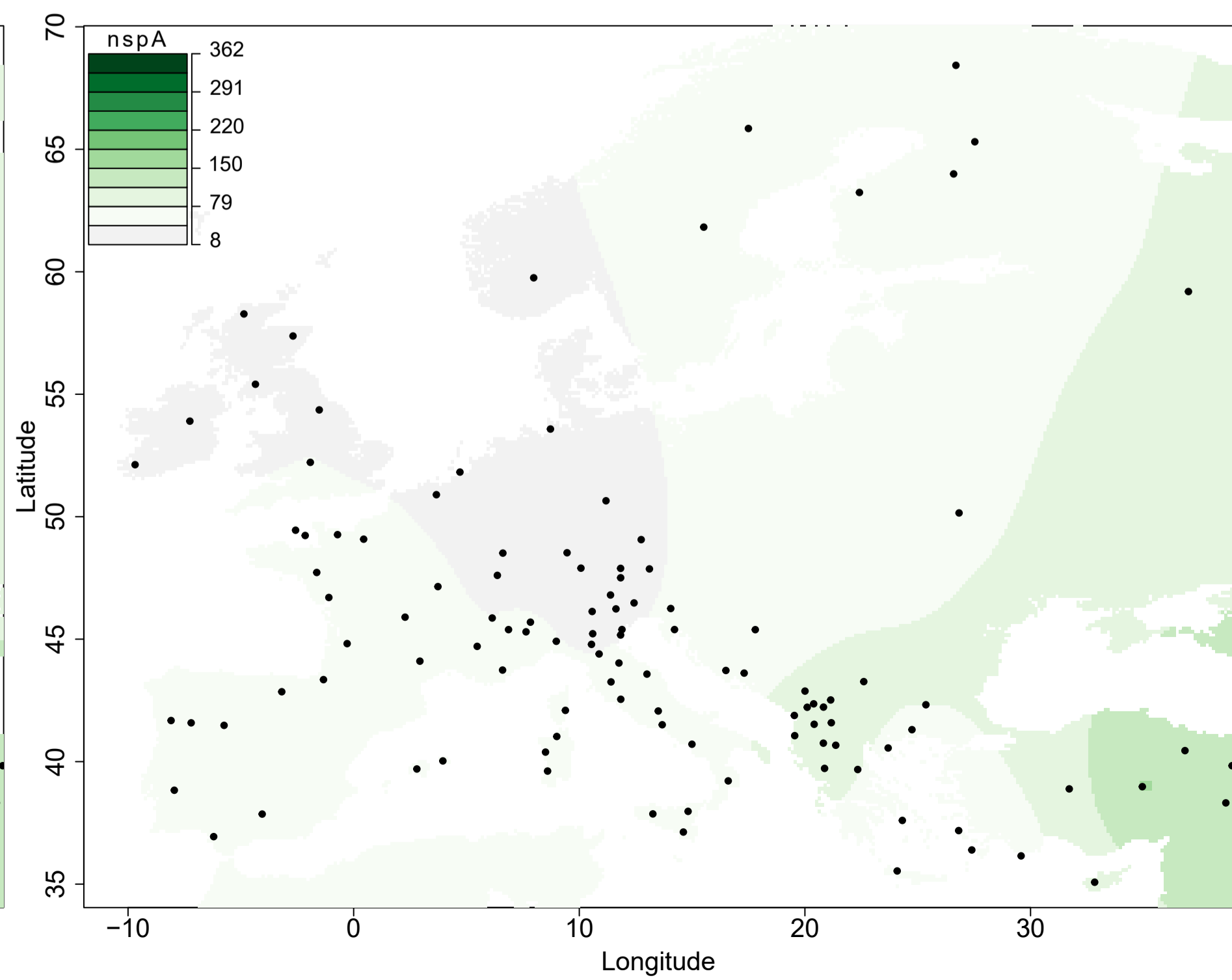

Supplement: Supplementary file 3 — Additional file 3: Figure S1. Tessellated projection. Spatial geographic presentation of the herein estimated diversity parameters (mA, AR, HE, HE(SNP), npA, nspA). [file 12711_2020_560_MOESM3_ESM.pdf]

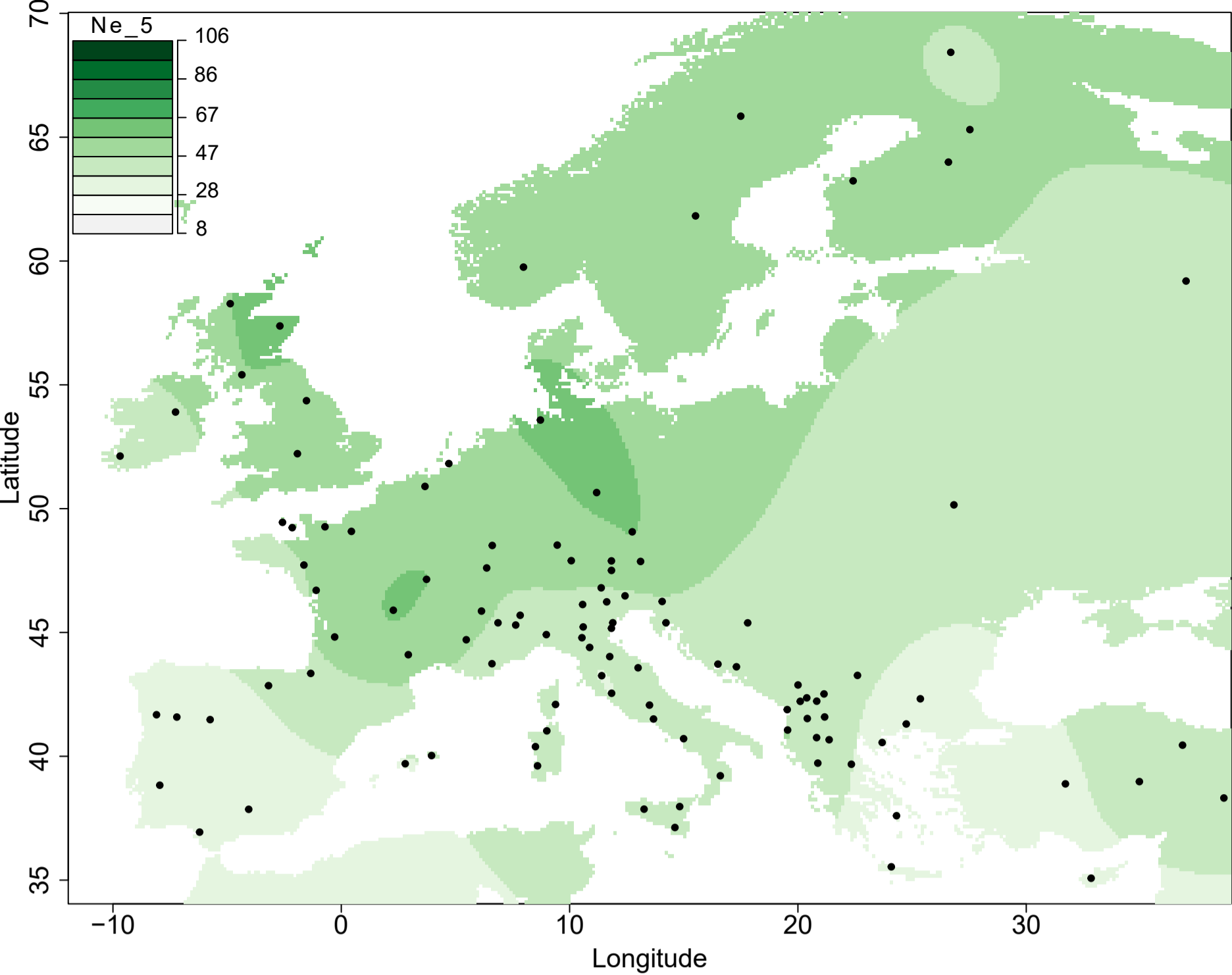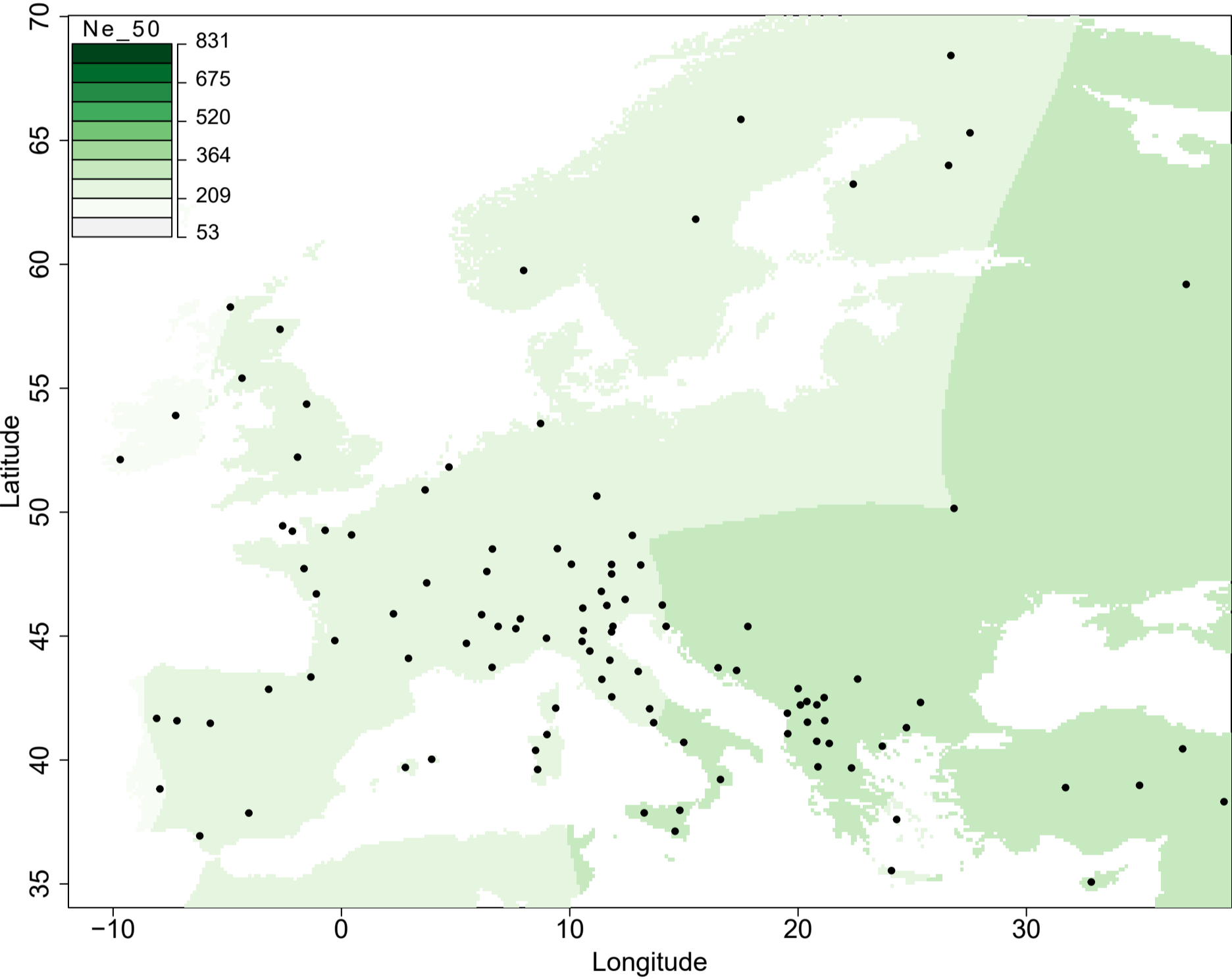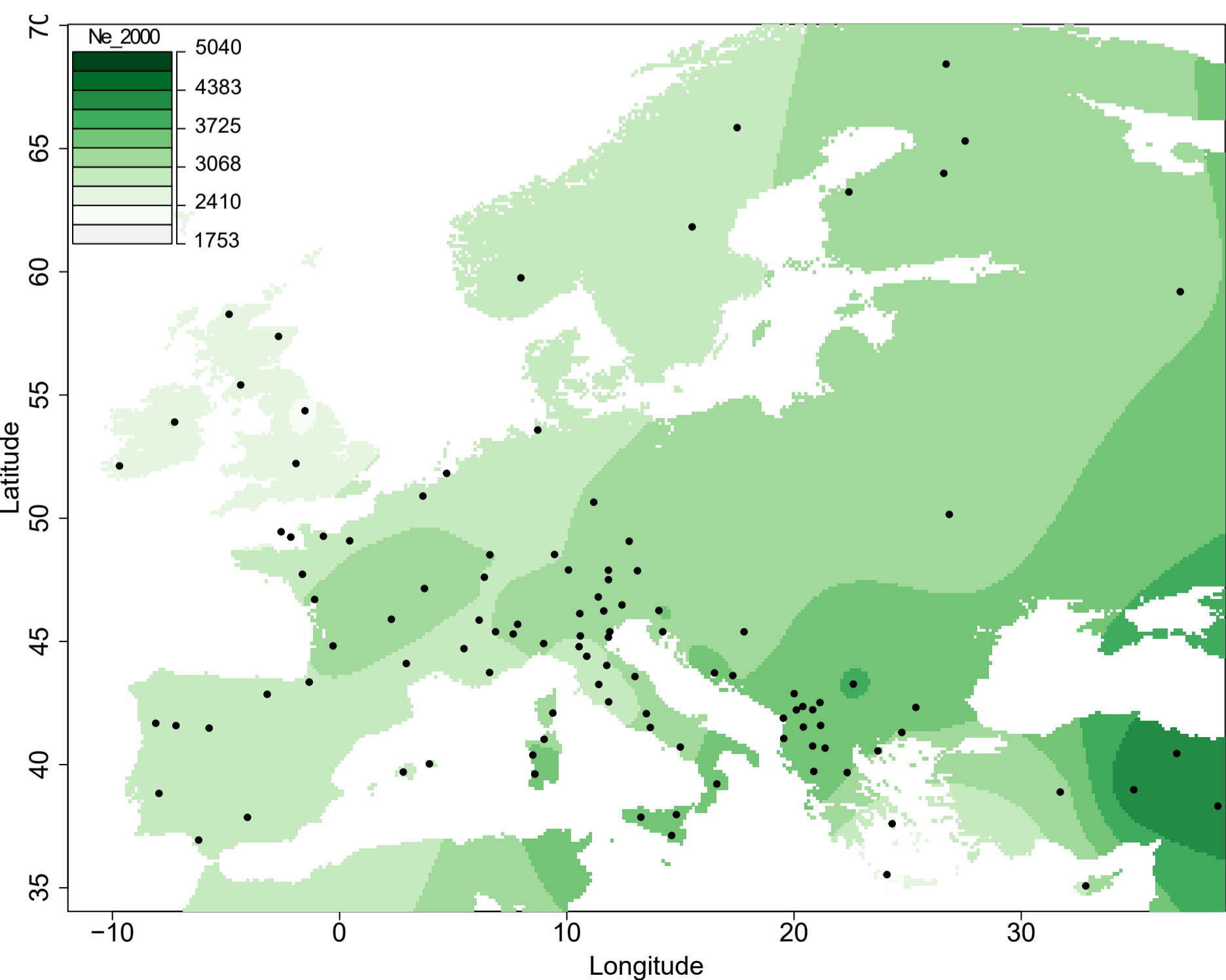

Supplement: Supplementary file 4 — Additional file 4: Figure S2. Tessellated projection. Spatial geographic presentation of the estimated effective population number (Ne5, Ne50, Ne2000). [file 12711_2020_560_MOESM4_ESM.pdf]

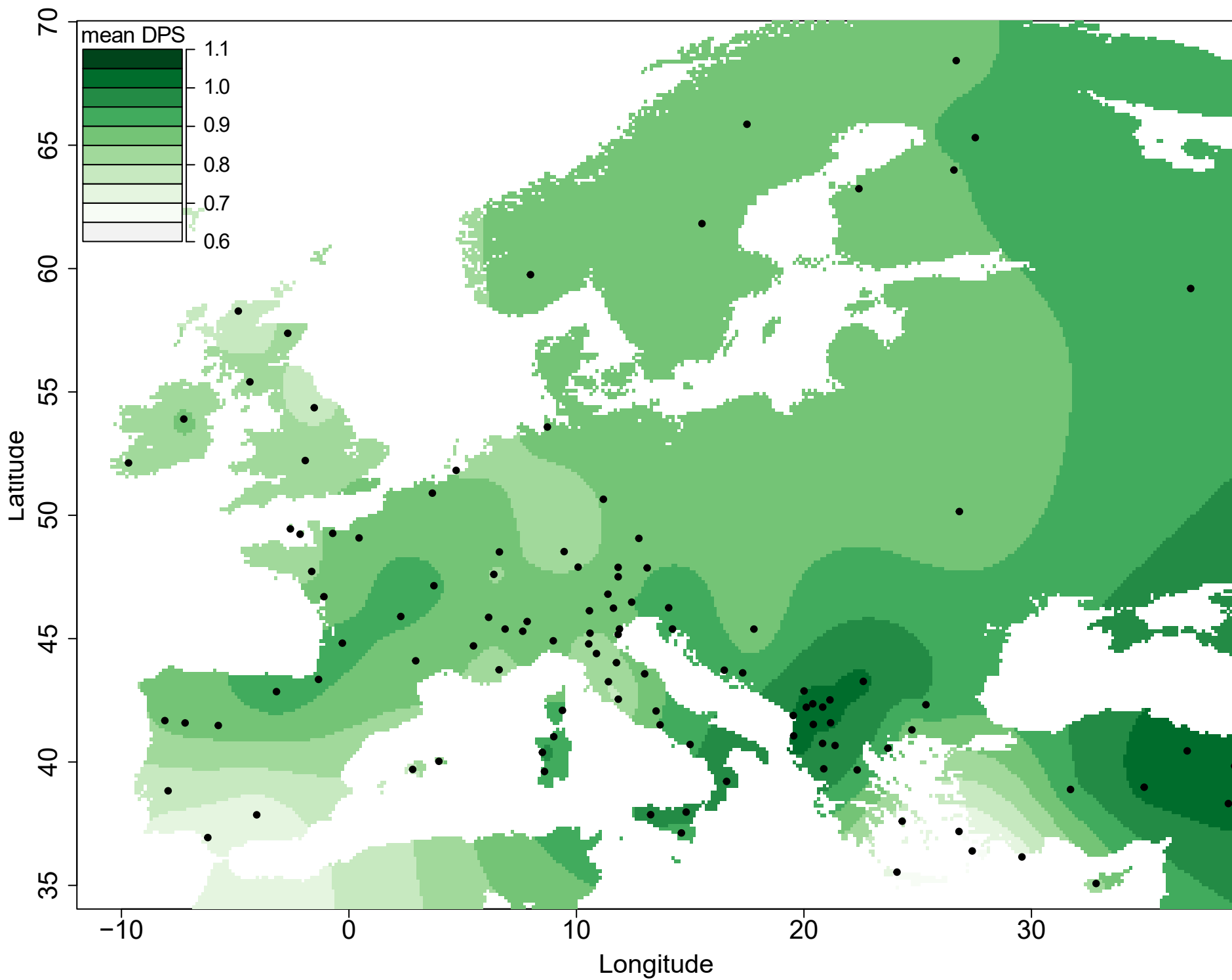

Supplement: Supplementary file 6 — Additional file 6: Figure S3. Tessellated projection. Spatial geographic presentation of the estimated allele sharing distance matrix (DPS) among breeds using multi-allelic SNP-blocks. [file 12711_2020_560_MOESM6_ESM.pdf]

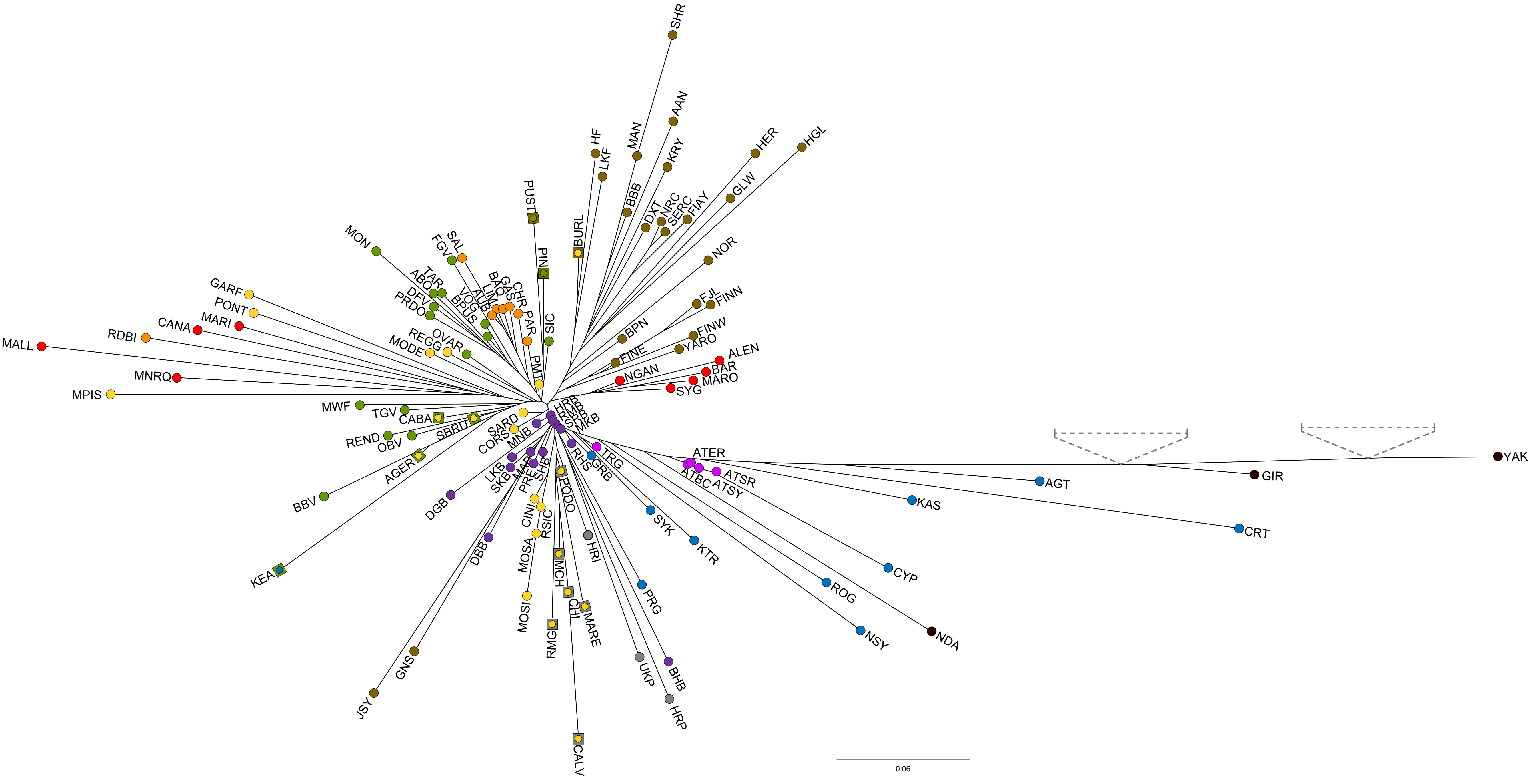

Supplement: Supplementary file 7 — Additional file 7: Figure S4. Phylogenetic tree. Neighbor-joining tree based on Nei’s genetic distance DA using multi-allelic SNP blocks. Mongolian yak (YAK) was used as a root. Dotted lines indicate the reduced length of YAK and GIR to improve visibility. Special square marks represent the influence of East-Podolian (grey), Alpine (green) and North-West (olive green) group. [file 12711_2020_560_MOESM7_ESM.pdf]
