# Supplementary material for: Genomic diversity and population structure of the indigenous Greek and Cypriot cattle populations
Source: Genet Sel Evol. 2020 Jul 29;52:43. doi: 10.1186/s12711-020-00560-8 (PMC7391618; doi:10.1186/s12711-020-00560-8)
Supplement: Supplementary file 8 — Additional file 8: Figure S5. Admixture analysis. Population structure of the 114 studied breeds/populations presented for each of K inferred ancestral population value, using a genome‐wide set of bi-allelic SNPs. All the analyzed individuals are presented as colored vertical bars, divided at most K segments and with proportional heights, according to their genotype membership. In the depicted plots (K = 2 to K = 26), a different color is presented for each cluster. From left to right within each group the presented breeds are as follows: Minor Asia: ATER, ATBC, ATSR, ATSY, TRG; Greece and Cyprus: CYP, AGT, CRT, NSY, GRB, KAS, KEA, PRG, ROG, SYK, KTR; South East Europe: RHS, MKB, SRB, PRE, RMB, SHB, DGB, DBB, MAB, LKB, SKB, MNB, BHB, HRB; East Podolian: HRI, HRP, UKP; Tyrrhenian: PODO, CINI, MOSI, RSIC, MOSA, SARD, SBRU, CORS, AGER, MARE, CHI, MPIS, CALV, MCH, RMG, GARF, PONT, MODE, CABA, REGG, PMT, BURL; Alpine: PRDO, OVAR, REND, BPUS, PUST, SIC, PIN, TGV, MWF, OBV, BBV, DFV, FGV, VOG, ABO, MON, TAR; France: RDBI, SAL, AUB, LIM, CHR, PAR, BAQ, GAS; Iberian: MNRQ, MALL, NGAN, CANA, MARI, ALEN, BAR, MARO, SYG; North West Europe: BPN, NOR, MAN, BBB, LKF, HF, GNS, JSY, HER, SHR, KRY, DXT, GLW, AAN, HGL, NRC, SERC, FJL, FIAY, FINE, FINW, FINN, YARO. [file 12711_2020_560_MOESM8_ESM.pdf]

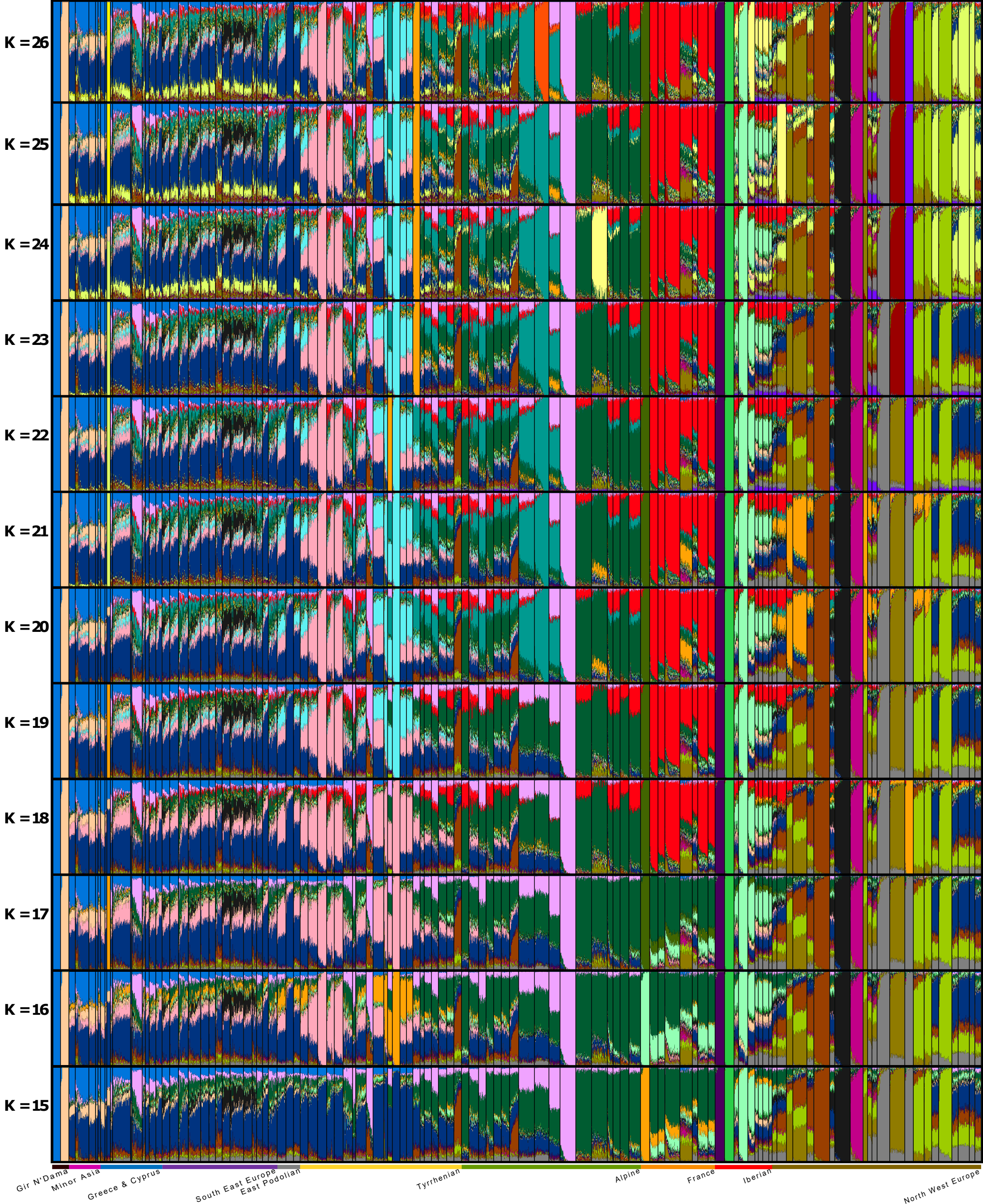
K = 26
K = 25
K = 24
K = 23
K = 22
K = 21
K = 20
K = 19
K = 18
K = 17
K = 16
K = 15
Gir N'Dama
Minor Asia
Greece & Cyprus
South East Europe
East Podolian
Tyrrhenian
Alpine
France
Iberian
North West Europe

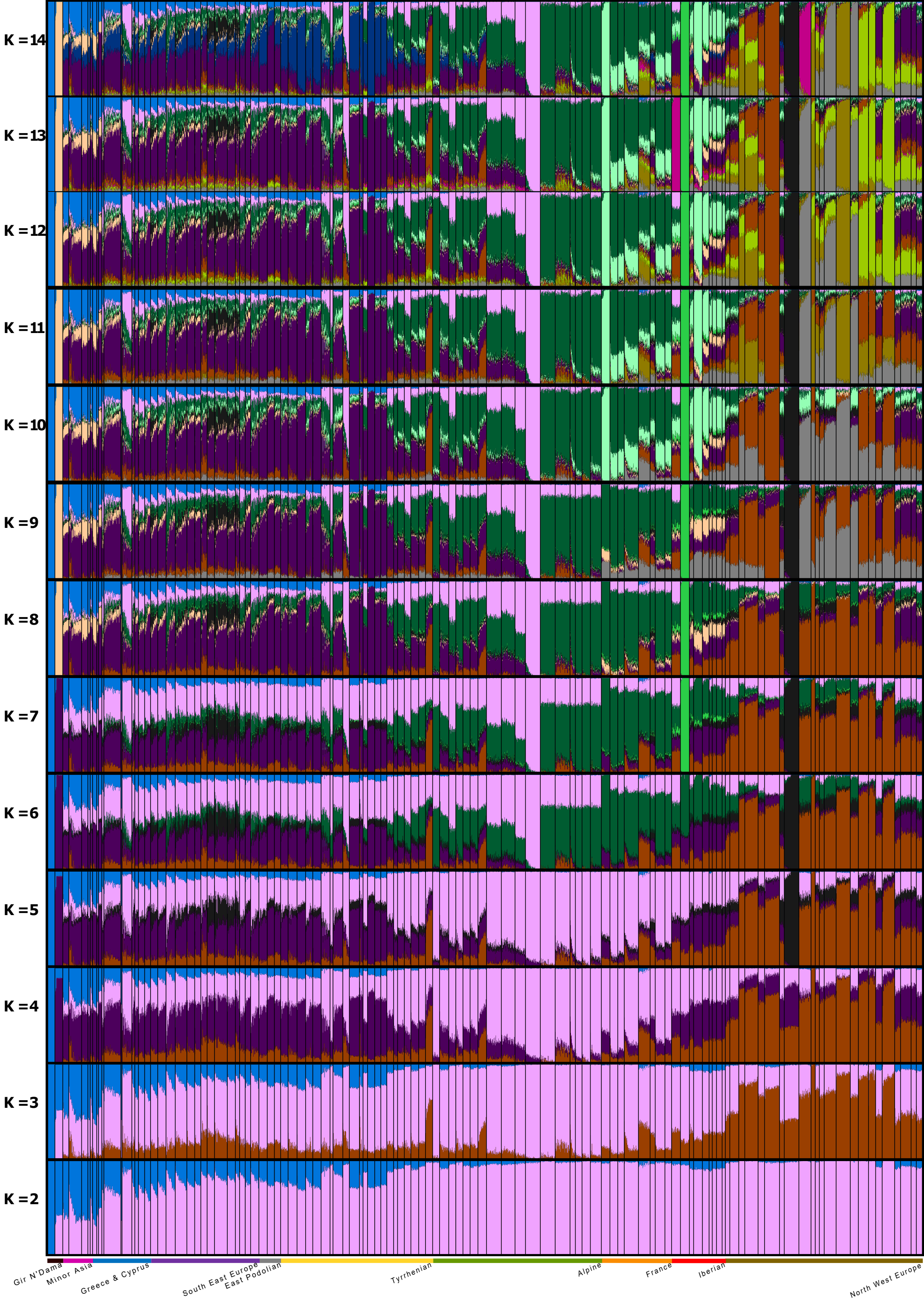
K = 14
K = 13
K = 12
K = 11
K = 10
K = 9
K = 8
K = 7
K = 6
K = 5
K = 4
K = 3
K = 2
Gir N'Dama
Minor Asia
Greece & Cyprus
South East Europe
East Podolian
Tyrrhenian
Alpine
France
Iberian
North West Europe
